# The Impact of Pre-Slaughter Stress on Beef Eating Quality

**DOI:** 10.3390/ani9090612

**Published:** 2019-08-27

**Authors:** Kate M.W. Loudon, Garth Tarr, Ian J. Lean, Rod Polkinghorne, Peter McGilchrist, Frank R. Dunshea, Graham E. Gardner, David W. Pethick

**Affiliations:** 1 School of Veterinary and Life Sciences, Murdoch University, Murdoch, WA 6150, Australia; 2 School of Mathematics and Statistics, The University of Sydney, Sydney, NSW 2006, Australia; 3 Scibus, Camden, NSW 2570, Australia; 4 Birkenwood Pty. Ltd., 431 Timor Rd, Murrurundi, NSW 2338, Australia; 5 School of Environmental and Rural Science, University of New England, Armidale, NSW 2350, Australia; 6 Faculty of Veterinary and Agricultural Sciences, The University of Melbourne, Melbourne, VIC 3010, Australia

**Keywords:** consumer eating quality, meat quality, beef, pre-slaughter stress

## Abstract

**Simple Summary:**

Consumer satisfaction is essential to the beef industry to ensure return protein purchasing. The Meat Standards Australia grading system has been pivotal in regulating the quality and consistency of meat palatability by creating objective measurements such as carcass characteristics, genetics systems, and production systems to predict consumer eating quality. One of the main objective measurements for carcass compliance is the ultimate pH of the *longissimus thoracis et lumborum*; however, recent research has demonstrated that pre-slaughter stress is eroding beef eating quality in pH compliant carcasses. Pre-slaughter mixing and transport was associated with lower eating quality in certain cuts. A two-week rest period at the abattoir prior to slaughter was beneficial for improving consumer sensory eating quality scores. Further research is required to determine if the muscle damage enzyme creatine kinase could be used commercially as an objective measurement to identify which cattle could benefit from a pre-slaughter rest period to improve beef quality.

**Abstract:**

The study evaluated the relationship between pre-slaughter stress, plasma biomarkers and consumer-evaluated eating quality of pasture raised beef cattle (n = 488). The design tested steer only, heifer only and mixed sex cattle with a comparison of direct kill versus a 14 day rest period in abattoir holding paddocks prior to slaughter. Experiment One sourced cattle from four farms and tested shipping and road transport. Experiment Two sourced cattle from four farms and tested a commercial saleyard pathway. The impact on treatment on untrained consumer eating quality scores were tested on five muscle groups, *m. psoas major, m. longissimus dorsi lumborum, m. biceps femoris, m. semitendinosis*, and *m. infraspinatus*. Across all muscles, a two-week rest period had the biggest improvement in sensory score. Mixed groups scored lower in the outside muscle than non-mixed groups. However, the mixing response was inconsistent in the eye round muscle and not significant in the other muscles. Plasma glucose and L-lactate indicated a marked acute stress response at slaughter with a small detrimental impact on consumer score. The muscle damage enzyme markers creatine kinase (CK) and aspartate aminotransferase (AST) were strongly associated with a lower meat quality score (MQ4). Neither β-hydroxybutyrate (βHB) nor non-esterified fatty acids (NEFA) were associated with MQ4, suggesting that fat mobilisation does not impact consumer sensory score.

## 1. Introduction

Producing a premium product which ensures a consistent, high-quality eating experience for the consumer is essential to the Australian beef industry to maintain the continued purchasing of beef. Australia introduced a beef-grading system, known as Meat Standards Australia (MSA), in 1998 to regulate the quality and consistency of meat palatability [1]. The MSA grading prediction of intrinsic eating quality is based on large-scale consumer sensory testing to quantify the impact of management, carcass phenotype measures and post-mortem processing on palatability [2]. Polkinghorne et al. [3] demonstrated that consumers purchasing a cut of meat with a predicted eating quality score are willing to pay significantly more per kilogram for product with higher eating grades. Furthermore, international consumer studies have demonstrated that willingness to pay for premium meat is universal, thus ensuring that the accurate prediction of eating quality remains vital to domestic and export markets [4,5,6].

While the MSA system is based on numerous factors including genotype, production system and carcass characteristics, the main objective measurement for meat quality in the current matrix is ultimate pH of the *longissimus thoracis et lumborum* (loin). One of the factors to qualify for MSA grading is the need for the loin muscle to have an ultimate pH (pHu) ≤ 5.7. Inadequate acidification and a high pHu are associated with the depletion of muscle glycogen stores prior to slaughter, which results from a combination of inadequate nutrition and pre-slaughter stress events. These carcasses are ineligible to receive a grade outcome under the MSA system [7,8,9]. However, studies by Warner et al. (2007) and Ferguson et al. (2008) reported that acute pre-slaughter stress negatively impacted consumer-assessed eating quality in pH compliant carcasses that had pHu (≤5.7). For example, acute pre-slaughter stress induced by electric cattle goads resulted in a reduced consumer acceptability of grilled loin steaks for tenderness, juiciness, flavour and overall liking, independent of muscle pH [10]. These findings were supported by Gruber et al. [11] who observed higher Warner Bratzler Shear Force (WBSF) in stressed cattle despite low pH of the muscles studied. However, Ferguson et al. (2008) noted that the effects of pre-slaughter stress on meat quality independent of pH are not always consistent, and there is a sparseness of data in ruminants which precludes a good knowledge and estimate of the cost of the effects [8].

MSA has developed pathways to minimise physical activity and emotional arousal in the pre-slaughter period. Certain pre-slaughter pathways which may maximise stress are penalised. For example, cattle sold through a saleyard prior to slaughter receive a five-point deduction in the resulting MSA meat quality score [1,12]. While these pathways were made with best practice and animal welfare in mind, the current literature is not conclusive on the impact of pre-slaughter stress on consumer-assessed eating quality. Ferguson [12] demonstrated only marginal differences in eating quality in cattle marketed through a saleyard compared to direct consignment, while Polkinghorne [13] showed no significant effects of extended transport time on objective meat quality. Additionally, Colditz [14] found no influence of social regrouping four, two or one week(s) prior to slaughter on consumer sensory meat perception. However, all three studies demonstrated large between-farm differences in eating quality even with seemingly similar on-farm backgrounds, such as British breed, young pasture raised cattle [12,13,14].

To date, consumer sensory studies have only evaluated the impact of pre-slaughter stress situations on the *longissimus thoracis et lumborum* [10,11,12,13,14]. This study was undertaken to examine the impact of pre-slaughter stress on consumer eating quality across five different cuts. We hypothesise that cattle subjected to simulated pre-slaughter stress will detrimentally impact consumer eating quality scores.

## 2. Materials and Methods

The detailed design for animal selection, treatment groups, trucking, shipping and lairage are described in the methodology by Loudon et al. [15] (Supplementary Materials).

Experiments were approved and monitored by the animal ethics committee at Murdoch University (Perth, Western Australia) with permit number R2839/16. Experimental design and protocols were established by an MSA pathways committee and intended to mimic Australian commercial conditions with the aim to provide industry-relevant recommendations.

Two experiments were conducted to assess the impact of pre-slaughter simulated stress and slaughter interval on consumer eating quality in pasture fed beef. The experiments utilised a total of 488 head of commercial beef cattle which were all 100% pasture raised from birth, of British breed (Hereford, Angus and Murray Grey), less than 24 months of age, and had not at any point in their life been administered antibiotics or growth promoting hormonal implants. Two geographical locations were used; Experiment One was conducted in King Island (n = 244), and Experiment Two was conducted in north western Tasmania (n = 240). In each location, cattle were sourced from four farms, 2 providing steers and 2 providing heifers to give an equal spread of sex.

The experiments were conducted in a replicated design, assessing three treatments in each location. Treatment 1 (mixing stress) and Treatment 3 (slaughter interval) were the same for both experiments. Treatment 1 evaluated social regrouping (mixing stress) via five different groups; (1) never mixed steers (NMS), (2) never mixed heifers (NMH), (3) mixed steers (MS), (4) mixed heifers (MH), and (5) mixed steers and heifers (MSex). Treatment 2 evaluated transport stress. In Experiment 1, the cattle travelled via boats and trucks to the processing plant. In Experiment 2, the cattle were auctioned through a saleyard prior to trucking to the processing plant. Treatment 3 evaluated slaughter interval. On arrival at the processing plant, a subset of cattle was slaughtered directly and compared against a cohort that were rested in pasture paddocks for two weeks prior to slaughter.

### 2.1. Blood and Muscle Sampling

Blood was collected from each animal during exsanguination into 1 × 9 mL lithium heparin Vacuette^®^ tubes (Greiner bio-one, Kremsmuenster, Austria), inverted gently seven times, and then placed into shaved ice in a cooler box prior to centrifugation at 2000 RPM for 15 min. Samples were separated within 2–4 h of collection and plasma transferred into 3 × 2 mL screw top tubes and frozen at −20 °C until further analysis. Plasma was analysed for glucose, L-lactate, non-esterified fatty acids (NEFA), magnesium (Mg), sodium (Na), chloride (Cl), β-hydroxybutyrate (βHB), haptoglobin (HP-T) and ceruloplasmin (CP) concentration and creatine kinase (CK), and aspartate aminotransferase (AST) activity. The detailed methodology is described by Loudon et al. [15]. All plasma samples were analysed within one month of collection.

A core sample (approximately 10 g) of M. longissimus thoracis at the quartering site was taken approximately 40 min after slaughter. A 10 cm stainless steel drill piece with a 1.5 cm core was used on a hand-held electric drill. All fat was removed from the muscle prior to placing in a 5 mL plastic screw top tube labelled with the body number. Samples were immediately placed on shaved ice in a cooler box then stored at −20 °C for a later analysis of glycogen, free glucose and lactate concentration. Detailed methodology is described by Loudon et al. [15], who utilised the muscle glycogen enzymatic methods of Chan and Exton [16] and the lactate methods of Noll [17].

### 2.2. Consumer Sensory Testing

Approximately 24 h after slaughter, 5 muscles were collected from the left side of each carcass: *M. psoas major* (tenderloin), *m. longissimus dorsi lumborum* (striploin), *m. biceps femoris* (outside), *m. semitendinosis* (eye round), and *m. infraspinatus* (oyster). Each cut was aged for either 7 or 21 days under vacuum packaging, after which steaks were prepared and frozen until tested by untrained consumer taste panels. Following the MSA tasting protocol, untrained consumers were selected for sensory evaluation to ensure that the final assessment of palatability would be based on the target consumer [18]. The protocol for primal sample collection and grill protocols for 25 mm thick steaks are described in Gee et al. [19] and summarised by Watson et al. [18]. Samples sourced from study carcasses were evaluated in 187 consumer sensory sessions, each utilising 60 untrained Australian consumers per session (n = 11,220 consumers). While the consumers were untrained, they were screened to include only people who preferred steak cooked to medium doneness, ate beef at least once a fortnight and were aged between 18 and 70 years old. Each consumer scored 6 test steaks on a scale of 0–100 for tenderness, liking of flavour, juiciness and overall liking, and they finally scored the steak as unsatisfactory, good every day, better than every day, or premium.

### 2.3. Statistical Analysis

Natural logarithm transforms were applied to plasma CK and AST activities as well as HP-T and CP concentrations, as these traits exhibited a high heterogeneity of variance. For these traits, all subsequent analyses were undertaken using the transformed data. Analysis was undertaken in R (v 3.5.3.) (Springer-Verlag, New York, USA), a linear mixed effects model was used to estimate the lme4 (v 1.1–21), post hoc tests were conducted using the emmeans package (v 1.4) (Springer-Verlag, New York, USA), and figures were created using the ggplot2 R package (v 3.1.1) (Springer-Verlag, New York, USA) [20,21,22,23].

Measurements taken in the two experimental phases were identical, with the key difference being the transport pathway from farm to abattoir, enabling data from both phases to be combined for statistical analysis and more rigorous consideration of the effect of different stressors, pathways to slaughter, and potential measures of stress.

A meat quality score (MQ4) was calculated using the weighted mean of consumer sensory scores as described by Watson et al. [18]. The weighting was: Tenderness 30%, juiciness 10%, flavour 30%, and overall liking 30%. The analysis tested muscle (oyster, eye round, outside, tenderloin, and striploin), days aged (7 and 21 days), and experimental treatment on MQ4.

The MQ4 of each muscle was analysed in a linear mixed effects model with the mixing group, transport type (boat or saleyard), slaughter interval (direct or rested), and days aged (7 and 21 days) as fixed effects, and the property of origin was used as a random term. Blood and muscle metabolites were tested covariates. Each metabolite was analysed individually, and each metabolite that was significant for an individual muscle tested together.

A linear mixed effects model was used to assess the impact of muscle and plasma metabolites, mixing transport, and slaughter interval on MQ4. The mixing group, transport type and slaughter interval were used as fixed effects, and the property of origin was a random term.

Due to the numerous factors that can have additive and complex interactions with stress (and from an industry standpoint to ensure greatest protection for the consumer with eating quality), for this analysis, the *p* value was viewed as a continuous measure of the compatibility between the data and the fitted model rather than a dichotomous value [24].

## 3. Results

### 3.1. Effect of Treatments on Eating Quality

**Figure 1:**
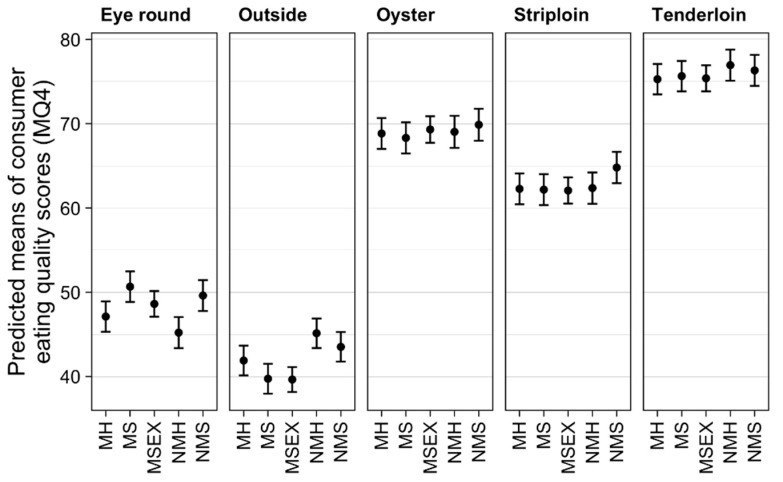
Effect of mixing treatment on individual muscle eating quality. The plots show predicted means with 95% confidence intervals where the results are averaged over the levels of sex, aging, transport method, and resting. The mixing treatments are: MH (Mixed Heifers), MS (Mixed Steer), MSEX (Mixed Sex), NMH (Never Mixed Heifers), NMS (Never Mixed Steers).

There was a significant mixing by muscle interaction (*p* < 0.001). While there were slight differences in the eating quality of individual muscles between mixing treatment groups, the response was not consistent across all muscles (Figure 1). Differences were found between mixing groups for the outside and eye round, but not for oyster, striploin or tenderloin.

The estimated MQ4 score was lower in the outside muscle for MH (−3.24 points, *p* = 0.028) and MS (−3.78 points, *p* = 0.05) compared to their unmixed counterparts (Figure 1). Similarly, the outside muscle MQ4 was lower in MS than NMH (−5.40 points, *p* = 0.05), while there was no difference between MH and NMS. Finally, the MQ4 of the outside muscle from MSex was lower than NMH (−5.49 points, *p* < 0.001) and NMS (−3.87 points, *p* < 0.001). The estimated MQ4 score was inconsistent in the eye round. NMH was lower than NMS (−4.40 points, *p* = 0.011) and MS (−5.45 points, *p* = 0.003).

**Figure 2:**
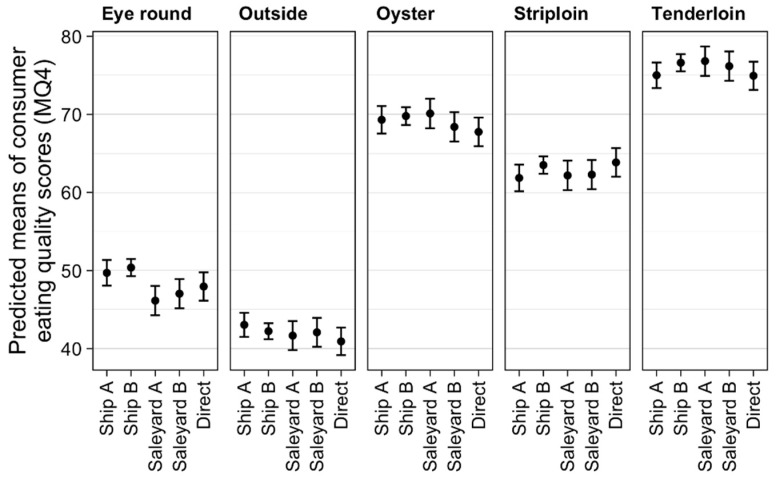
Effect of transport treatment on individual muscle eating quality. The plots show predicted means with 95% confidence intervals where the results are averaged over the levels of sex, aging, mixing method, and resting.

The transport treatment was significant through its interaction with muscle (*p* < 0.01). The estimated MQ4 score was lower in the eye round for Saleyard A (−4.23 points, *p* = 0.002) and Saleyard B (−3.34 points, *p* = 0.023) compared to Ship B (Figure 2).

**Figure 3:**
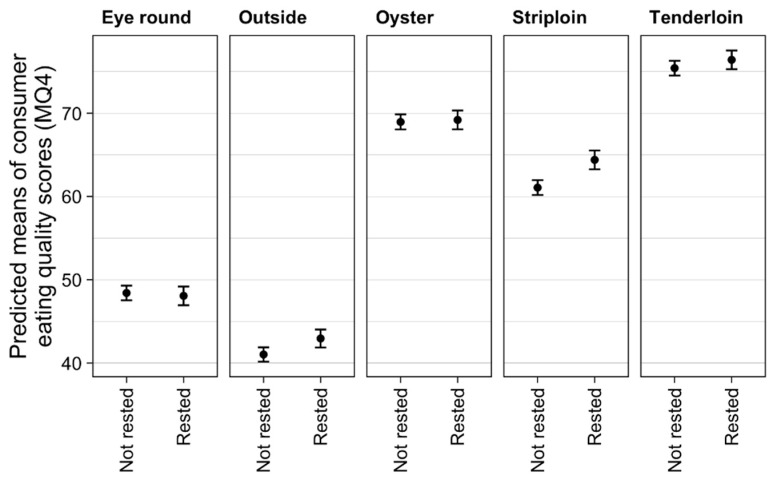
Effect of resting treatment on individual muscle eating quality. The plots show predicted means with 95% confidence intervals where the results are averaged over the levels of sex, aging, mixing and transport method.

The resting treatment had a significant effect on eating quality (*p* < 0.009). Across all muscles, there was an improvement in MQ4 in rested cattle compared to direct kill (60.2 vs. 59.0 points, *p* < 0.009). However, within cuts, the resting effect was only significant in the striploin, where there was a 3.3 point increase in MQ4 in rested cattle (*p* < 0.01, Figure 3).

There was no effect of aging on eating quality in the oyster and tenderloin, although aging did increase eating quality in the striploin (+4.3 points, *p* < 0.001), outside (+2.0 points, *p* < 0.001), and eye round (+1.3 points, *p* = 0.019).

### 3.2. Associations between Blood Metabolites and Eating Quality

Table 1 shows the raw data means and standard deviation for plasma metabolite and muscle glycogen concentrations. The high glucose and L-lactate concentrations observed suggest a strong adrenergic response that was stimulating glycogenolysis and glycolysis in the cattle at slaughter. Muscle damage enzyme CK activity also had a marked response, and the range observed suggests certain cattle had more muscle damage.

Blood and muscle metabolites were associated with consumer eating quality scores (MQ4) (Table 2).

There was a negative association between plasma L-lactate, glucose concentrations, and MQ4 in most cuts. Only the relationship with L-lactate and MQ4 for the outside and eye round cuts was significant. A 1 mmol/L increase in plasma L-lactate concentration was associated with a decrease in MQ4 by 0.35 units in the outside and by 0.23 in the eye round. There was no significant association between plasma glucose concentrations and the MQ4 of any of the cuts.

Plasma βHB and NEFA concentrations were positively associated with MQ4. A 0.1 mmol/L increase in plasma βHB concentration was associated with a 0.76 point increase in MQ4 in the eye round and a 0.36 mmol/L increase in the tenderloin. A 0.1 mmol/L rise in plasma NEFA was associated with an increase in MQ4 of 0.44 points in the eye round and by 4.38 in the oyster.

Plasma magnesium was positively associated with the striploin MQ4 (*p* < 0.01, Table 2)—as concentrations increased by 0.1 mmol/L, MQ4 improved by 0.99 points.

Plasma sodium was only significantly associated with the MQ4 of the eye round (*p* < 0.05, Table 2). A 1 mmol/L increase was associated with a decrease in MQ4 by 0.21 points; however, no other cuts were significantly associated, nor was there any significance with the dehydration marker chloride.

**Figure 4:**
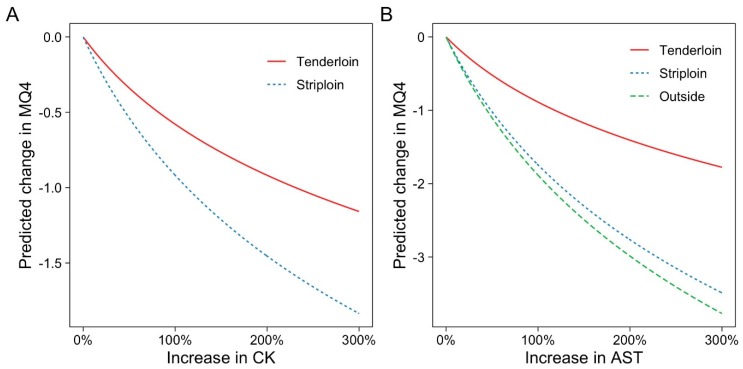
Associations of aspartate aminotransferase (AST) and creatine kinase (CK) with MQ4. Only the coefficients with *p*-values less than 0.1 are shown.

Muscle-derived plasma enzymes CK and AST were negatively associated with MQ4. As CK and AST activity increased, MQ4 decreased in the striploin and tenderloin (Table 2). Increased AST was also associated with a lower MQ4 of the outside. The log of these bloods were used in the model—thus, the response on MQ4 is non-linear (Figure 4). An increase in CK was associated with an MQ4 decrease in the striploin of a 1% increase by a 0.01 point decrease; a 10% increase by a 0.13 decrease; a 100% increase by a 0.92 point decrease; and a 200% increase by a 1.45 point decrease in MQ4. There was no significant interaction between days aged and CK except in the eye round (*p* = 0.028), where there was an increased MQ4 at 21 days.

## 4. Discussion

Mixing and transport stressors had minimal impact on predicted means for consumer eating quality, in contrast to our hypothesis. The overall pattern of MQ4 scores for each cut was consistent with current MSA predictions [1], with the tenderloin rated the highest, followed by oyster and striploin, whilst eye round and outside were considered to be the toughest cuts.

The observation that a two-week rest period prior to slaughter improves MQ4 has not been previously reported. Across all five cuts, there was a positive response to resting, although this was only significant in the striploin. This result suggests that the cattle were experiencing stress when taken to immediate slaughter, but that the mixing and transport stressors may not have provoked a large enough difference between the experimental groups. Resting after acute stress is crucial for muscle glycogen repletion and is known to take over a week to replete after stressful incidents in cattle [29,30]. Loudon et al. [15] demonstrated that a two-week rest period was enough to replete muscle glycogen concentration and that cattle rested were 40% less likely to be non-compliant for ultimate pH. Muscle glycogen concentration had a positive but non-significant association with MQ4 (Table 2). It is possible, therefore, that initial transport and diet or holding conditions combined to ensure that the repletion of muscle glycogen was not sufficient to influence MQ4 in these cattle. Stewart et al. [31] demonstrated that there was an association between lamb loin WBSF and kill order, where lambs slaughtered on the same day but with an increasing kill order from 0 to 300 resulted in a 12% increase in shear force. Plasma indicators of stress measured in that study had no association with WBSF [31]. While kill order was not analysed in this experiment, our result suggests that duration of pre-slaughter stress exposure does impact MQ4 and that a rest period may be beneficial in improving eating quality in consigned groups exposed to severe stress, independent of muscle glycogen concentrations. Loudon et al. [15] found that plasma CK was inversely correlated with ultimate pH and muscle glycogen concentration, and it was the only plasma indicator with a consistent association to slaughter interval and a pre-slaughter rest period. The CK concentrations in this experiment were negatively associated with MQ4, which suggests that this blood biomarker may be a useful indicator of cattle that may benefit from a pre-slaughter rest period to improve eating quality.

The response of mixing cattle during transport on MQ4 score was inconsistent. Mixing was most detrimental in the outside cut, as the never mixed cattle (NMS and NMH) aggregated into one group with similar MQ4, and the mixed cattle (MSex, MS and MH) aggregated into another group which had lower MQ4 scores. However, there was no significant difference in the outside between MH and NMS, so the sex by mixing groups could not be entirely segregated into different eating quality groupings. The mixing effect on the eye round was an unexpected and contradictory result, such that the NMH had the lowest predicted eating quality of all groups. In the higher quality cuts, non-mixed cattle had slight improvements in MQ4 in the tenderloin, but for the striploin, the response was only seen in the steers with no response in the oyster muscle. Colditz et al. [14] evaluated the social regrouping of feedlot cattle prior to slaughter and found no change in sensory eating quality scores in the striploin with pre-slaughter mixing, even though one replicate regrouped one week prior to slaughter had increased compression scores. The authors concluded that property of origin had the greatest difference on eating quality, irrespective of mixing treatment. However, as the Colditz et al. [14] experimental cattle were obtained from only two properties, it is difficult to draw strong conclusions without sufficient experimental power.

Transport pathway had no effect on eating quality, in contrast to our hypothesis. However, there was a difference in eating quality in the eye round cut, where cattle transported by one ship from Experiment One had improved MQ4 compared to the saleyard pathway of Experiment Two. However, there was no difference in MQ4 between the two boats or the gold standard of direct consignment from farm versus saleyard. Ferguson et al. [12] concluded that in comparison to direct consignments, selling cattle through a saleyard had a small detrimental impact on MQ4. This overall difference was variable, and the effect of property of origin was far greater. Property of origin was included in our analysis as a random effect and likely did explain some of the variation in MQ4. The marked difference in MQ4 attributable to property of origin is important to pursue in further research to determine sources of the variance. As the effect of property of origin is not a satisfactory predictor for beef grading, it will be unable to be included in a production level model; however, factors that influence the variation attributable to property may be suitable for inclusion.

While individual plasma biomarkers displayed limited associations with MQ4, it is clear that physiological changes reflected in the biomarkers are associated with eating quality. Plasma enzymes CK and AST had the most significant association with MQ4, where an increase of enzyme activity had decline on MQ4 for all cuts except the oyster. This finding is in contrast with previous studies on plasma CK and tenderness. Gruber et al. [11] observed that plasma CK at slaughter was correlated with increased post transport agitation in behavioural scoring but had no impact on WBSF. However, there was a marked difference in transport time with cattle trucked only for 64–90 min, and a lower CK mean was observed (571.4 IU/L). Thus further investigation is warranted to determine if there is an association with transit time, muscle damage and meat quality. In Merino lambs at slaughter, Stewart et al. [31] observed that increasing plasma CK activity was associated with a reduced WBSF; however, the effect was not significant when corrected for pHu, which increased WBSF by 37% as pHu increased from 5.4 to 6.2. In contrast, in this study, there was no association between pHu and MQ4. Hence, this did not account for the association between CK and MQ4.

There are a number of possible mechanisms explaining the link between CK and MQ4. Firstly, CK may be reflecting differences in proteolysis. Supporting proteolysis as a likely mechanism was fact that the association between MQ4 with circulating CK in this study was most pronounced in the striploin, a muscle with a high proteolytic rate [32]. Alternatively, for the majority of cuts, there was no significant interaction between aging and CK, except in the eye round, where there was a slight tendency for MQ4 to increase with aging. Other studies have shown a link between pre-slaughter transport, exercise, and increasing CK [11,33,34,35,36]. In lambs, exercise has been shown to increase post-mortem proteolysis, although in this case, it was not sufficient to impact on WBSF tenderness [37,38]. Secondly, CK may be reflecting variation in pH decline. The catalytic enzyme CK is important for phosphorylating creatine phosphate to regenerate ADP to ATP during the early post-slaughter stage, thus delaying lactate production from glycolysis and subsequent muscle acidification [39,40]. The inactivation of creatine kinase and reduction in creatine phosphate influenced pH decline by reducing the delay phase [39,40,41,42]. This accelerated glycolysis may have pre-disposed these cattle to heat toughening conditions, which would reduce the aging potential and increase drip loss from the meat [43,44]. This has been demonstrated in pork, where denaturation of creatine kinase is associated with poor eating quality via a high drip loss and a pale, soft and exudative end product [45,46]. Further research into the impacts of CK levels on beef drip loss, proteolysis, and pH decline is required.

Acute stress biomarkers glucose and lactose were negatively associated with MQ4, although the effect was only significant for L-lactate, and the response varied across cuts. On average, a 1 mmol/L increase in L-lactate had a 0.35 predicted point deduction (*p* = 0.015) on the outside MQ4 score and a 0.23 point deduction (*p* = 0.087) in the eye round. Interestingly, there was a lower association of L-lactate with the traditionally higher quality cuts of the tenderloin and striploin. The average coefficient estimate for impact of L-lactate on MQ4 across all five cuts was −0.16 points, which, when based on the mean experimental L-lactate of 13.25 ± 3.17 mmol/L, would equate to a 2.12 unit deduction in MQ4. Overall, this a small effect and probably not detectable by consumers [47]. In contrast, hormone growth promotants have, on average, a five point deduction in eating quality [48], and modified atmosphere packaging had a 10–12 point deduction [49]. Polkinghorne [13] examined the effects of extended duration road transport on beef eating quality and found no association between plasma L-lactate and MQ4 but did find a negative relationship with glucose. This effect was most pronounced in lean carcasses with a high ultimate pH [13]. Contrary to our results, Warner et al. [10] found that a higher plasma L-lactate concentration in cattle electrically prodded 15 min prior to slaughter was a good predictor of consumer eating quality in ultimate pH compliant carcasses. This impact of L-lactate on meat quality, independent of pH, was supported by Gruber et al. [11] who demonstrated increases in plasma L-lactate taken at slaughter was correlated with on-farm and post-transport adverse behavioural scores, as well as an increase in WBSF in pH-compliant carcasses (pH < 5.8). Plasma glucose at slaughter was associated with increased post transport agitation behavioural scores and increased WBSF, independent of pHu [11]. In lambs, ultimate pH was positively associated with slaughter glucose and L-lactate; however, these metabolites had no association with WBSF [31,50]. Interestingly, there was an association with WBSF and increasing kill order in lambs, and a linear association of glucose and L-lactate had a linear association with kill order, which suggests that pre-slaughter stress may be reducing meat quality [31,50,51]. The discrepancies among these studies highlight certain pitfalls in interpreting these metabolites in the context of an objective measurement of stress on meat quality. Plasma half-lives of glucose and L-lactate are typically short, and their concentrations can be affected by multiple mechanisms [52]. Hyperglycaemia after transport or surgical stress has been shown to reduce in five-to-six hours [53,54]. However, in a serial blood samples taken every five hours after mixing stress in bulls, Warris et al. [36] demonstrated no significant change in blood glucose overnight. While the plasma L-lactate half-life in healthy cattle after varying levels of exercise is unknown, the half-life can be as short as 60 min in humans [55]. Loudon et al. [15] found no difference in plasma L-lactate or glucose between groups subjected to varying mixing or transport. Hence, the farm-gate to slaughter interval may be too long to distinguish between stressor intensities and may only reflect acute adrenergic surges at the processing plant.

The associations between plasma sodium and chloride that can reflect hydration states and MQ4 were inconsistent and significant only for sodium in one cut. Water deprivation typically results in total body water loss and increased plasma osmolality, hypernatremia and hyperchloraemia. The range of sodium and chloride in the study indicates that certain cattle were hypertonically dehydrated—however, the mean values lie within normal expected physiological ranges. In slaughter, lambs’ sodium had a positive association with pHu in only one of four groups and had no association with WBSF [31,50]. Additional markers such as haematocrit and urine specific gravity would have provided a more thorough evaluation of haemoconcentration and dehydration. Nonetheless, these results support those of Polkinghorne, who found no association between urine specific gravity and sensory meat quality [13].

Plasma NEFA and βHB, which can indicate feed deprivation, were positively related to MQ4 across all five cuts. This finding contrasts with the evaluation of biomarkers in slaughter lambs, where there was a significant association between βHB and pHu but not NEFA, and neither metabolite was associated with WBSF [31,50]. Lipolysis and consequent elevation in NEFA can be a result of a negative energy balance and adrenergic stimulation. Other studies have demonstrated that increases in NEFA concentrations are predominately driven by fasting, with catecholamine release resulting in smaller increases of NEFA [50,56]. Regardless of the source of NEFA increase, our result suggests that fasting did not adversely impact on MQ4 and, when evaluated with glucose and L-lactate, nor did acute adrenergic stress. This result supports the findings of Polkinghorne et al. [13], who found no association between circulating βHB, NEFA or L-lactate concentrations, and consumer meat quality sensory scores.

Plasma magnesium was positively associated with MQ4 across all five cuts, although the association was only significant in the striploin. Pre-slaughter magnesium supplementation in pigs [57] and sheep [58] has been associated with lower ultimate pH and meat quality, presumably by reducing neuromuscular stimulation and catecholamine release from the adrenal medulla. The relationship with magnesium and catecholamines is cyclical in nature, as catecholamine release causes a shift of magnesium from the extracellular into the intracellular fluid and the resultant decreased circulating magnesium induces a type of stress and increased catecholamine secretion [59,60,61]. The association between plasma magnesium and MQ4 is small, and as muscle glycogen was not related to MQ4, it suggests the mechanism of magnesium is not through a protective effect on glycogen. The range of plasma magnesium concentrations in the cattle suggest that some were hypomagnesaemic [25], a condition associated with muscle fasciculations, tremors and impaired cell membrane integrity. Human studies have demonstrated that magnesium supplementation prior to exercise results in a lower creatine kinase activity [62,63]. Further research is required to determine if hypomagnesaemia compounds exertional rhabdomyolysis in cattle and if supplementation can protect muscle function in transport.

## 5. Conclusions

The study identified that mixing in saleyards and mixing during transport was associated with lower eating quality in certain cuts. A two week resting prior to slaughter improved consumer sensory eating quality score. Further research into rest periods in different conditions and economic modelling would be useful to determine the viability of implementing this commercially.

The plasma metabolites varied within treatment groups. There are many limitations in studying the effects of stress and MQ4 based on bloods collected at exsanguination. However, from a large-scale commercial experiment, this is the practical point of collection in both experimental and implementation scenarios. The plasma metabolites reflected an accumulation of chronic and acute stress events. However, as the acute effects are temporally associated with slaughter, these may mask subtler chronic effects. The study demonstrated that acute stress markers glucose and L-lactate become very elevated at point of slaughter. Only L-lactate was associated with a negative response in MQ4. There was a surprising positive association of βHB and NEFA with MQ4. While the effects are small, this suggests that fat mobilisation within the limits of this study were not detrimental to MQ4 and raises the possibility that cattle that are capable of mobilising body lipid and are able to cope better with the stressors incurred in transport, mixing and slaughter.

Plasma CK was the biomarker most correlated with eating quality, being inversely correlated to ultimate pH, muscle glycogen concentration and consumer eating scores. The mechanism of action in the association between CK and eating quality is unclear. The finding may reflect muscle damage. However, further research is required to determine if CK could be a useful objective measurement pre-slaughter to improve MSA compliance and eating quality.

## Figures and Tables

**Table 1:** Descriptive statistics of plasma metabolites taken immediately at slaughter and m. longissimus lumborum glycogen.

Variable	Mean	SD	Min	Max	Published Normal Basal Concentrations
Glucose (mmol/L)	6.83	1.22	3.69	14.51	1.9–3.8 ^a^
Lactate (mmol/L)	13.25	3.17	5.78	23.48	0.6–2.2 ^a^
CK (IU/L)	870.15	1056.29	113.30	9384.90	35–280 ^a^
AST (IU/L)	122.14	62.06	55.57	636.17	78–132 ^a^
βHB (mmol/L)	0.26	0.14	0.03	0.85	0.35–0.47 ^a^
NEFA (mmol/L)	0.46	0.26	0.08	1.52	<0.4 ^a^
Magnesium (mmol/L)	0.79	0.10	0.48	1.10	0.74–1.10 ^a^
Sodium (mmol/L)	147.84	3.45	125.70	160.80	132–152 ^a^
Chloride (mmol/L)	97.96	3.33	91.40	119.50	95–110 ^a^
Haptoglobin (mg/mL)	0.31	0.40	0.01	3.24	0.0–0.2 ^b^
Ceruloplasmin (IU/L)	95.99	42.34	20.00	285.00	15–68
Muscle Glycogen (g/100 g)	1.08	0.24	0.31	1.78	

^a^ [25] ^b^ [26,27] ^c^ [28].

**Table 2:** Coefficient estimates for plasma with meat quality score (MQ4) for five diferent muscles in all cattle, King Island and Tasmania. *P* value is in brackets. Coefficients with *p*-values less than 0.1 are highlighted are in bold.

Variable	Eye Round	Outside	Oyster	Stiploin	Tenderloin
Glucose (mmol/L)	−0.114 (0.759)	−0.422 (0.272)	−0.427 (0.139)	−0.323 (0.356)	0.119 (0.584)
Lactate (mmol/L)	−0.231 (0.087)	−0.349 (0.015)	−0.100 (0.350)	−0.090 (0.502)	−0.024 (0.767)
CK (log)	−0.461 (0.429)	−0.444 (0.476)	0.217 (0.669)	−1.323 (0.041)	−0.835 (0.017)
AST (log)	−0.116 (0.925)	−2.715 (0.037)	1.505 (0.123)	−2.511 (0.034)	−1.281 (0.088)
βHB (mmol/L)	7.590 (0.010)	3.839 (0.225)	3.949 (0.096)	1.793 (0.538)	3.625 (0.049)
NEFA (mmol/L)	4.379 (0.013)	1.973 (0.286)	2.306 (0.093)	1.134 (0.495)	0.355 (0.734)
Magnesium (mmol/L)	5.826 (0.136)	3.683 (0.378)	3.249 (0.297)	9.921 (0.008)	0.426 (0.859)
Sodium (mmol/L)	−0.211 (0.066)	−0.101 (0.419)	−0.057 (0.539)	−0.011 (0.923)	−0.048 (0.501)
Chloride (mmol/L)	0.007 (0.951)	0.051 (0.703)	−0.019 (0.846)	−0.008 (0.951)	−0.035 (0.648)
Haptoglobin (log)	−0.036 (0.928)	0.128 (0.757)	−0.376 (0.231)	−0.411 (0.294)	−0.212 (0.384)
Ceruloplasmin (log)	−0.573 (0.627)	0.037 (0.975)	−0.263 (0.767)	−0.624 (0.623)	0.526 (0.449)
Muscle Glycogen (g/100 g)	−0.306 (0.897)	3.083 (0.213)	−0.317 (0.868)	2.728 (0.247)	−1.094 (0.449)

## Supplementary Materials

The following are available online at https://www.mdpi.com/2076-2615/9/9/612/s1, The full methodology is available in Loudon et al. [15].
