# Supplementary material for: The Impact of Pre-Slaughter Stress on Beef Eating Quality
Source: Animals (Basel). 2019 Aug 27;9(9):612. doi: 10.3390/ani9090612 (PMC6769826; doi:10.3390/ani9090612)
Supplement: Supplementary file 1 [file animals-09-00612-s001.pdf]

animals MDPI

Article

The use of biochemical measurements to identify pre-slaughter stress in pasture finished beef cattle

Kate M. W. Loudon[1*], Garth Tarr[2], David W. Pethick[1], Ian J. Lean[3], Rod Polkinghorne[4] and Maddison Mason[1], Frank R. Dunshea[5], Graham E. Gardner[1] and Peter McGilchrist[1,6]

[1] School of Veterinary and Life Sciences, Murdoch University, Murdoch, WA, 6150 Australia; d.pethick@murdoch.edu.au (D.W.P.); maddison.mason@live.com.au (M.M.); g.gardner@murdoch.edu.au (G.E.G.);
[2] The University of Sydney, School of Mathematics and Statistics, Sydney, NSW 2006, Australia; garth.tarr@sydney.edu.au
[3] Scibus, Camden, NSW, 2570 Australia; ianl@scibus.com.au
[4] Birkenwood Pty. Ltd, 431 Timor Rd, Murrurundi, NSW 2338, Australia; rod.polkinghorne@gmail.com
[5] Faculty of Veterinary and Agricultural Sciences, The University of Melbourne, VIC 3010, Australia; fdunshea@unimelb.edu.au
[6] School of Environmental and Rural Science, University of New England, Armidale, NSW, 2350 Australia peter.mcgilchrist@une.edu.au
* Correspondence: k.loudon@murdoch.edu.au; Tel.: +61-8-9360-6000

Received: date; Accepted: date; Published: date

Simple Summary: Producing a product that delivers a consistently high-quality eating experience is paramount to the Australian beef industry to ensure consumer satisfaction and return protein purchasing. The importance of minimising pre-slaughter stress in cattle for animal welfare and meat quality is well understood by the industry however there currently exists no objective measurement of detecting which cattle are at greatest risk of producing poor quality meat. A pre-slaughter measurement would enable the beef industry to detect at risk cattle and implement an intervention strategy prior to slaughter. Muscle damage enzyme creatine kinase was the plasma biomarker most correlated with meat quality and a two week rest period prior to slaughter was beneficial at improving quality. Further research is required to determine on a commercial scale the usefulness of creatine kinase as an objective measurement and the cost benefit of a two week rest period for the industry.

Abstract: The study considers the relationship between pre-slaughter stressors and plasma biomarkers in 488 pasture raised cattle across two experiments. The design aimed to test groups consisting of steer only, heifer only, and mixed sex cattle under direct kill versus rested (14 days in abattoir holding paddocks) protocols. In Experiment One, cattle were sourced from 4 farms, transported by trucks and ships, on the same day. In Experiment Two, cattle were sourced from 4 farms, where a comparison was made between marketing via two commercial saleyards or direct farm gate consignment to abattoir. Blood samples were collected at exsanguination for subsequent analyses and relation to meat quality attributes. Muscle damage, as indicated by CK, is the metabolite most correlated to ultimate pH and muscle glycogen concentrations. A two week rest period is effective at lowering this enzyme and improving muscle glycogen concentration. Despite the cattle subjected to a range of stress inducing treatments, we find that plasma biomarkers alone appear insufficient for use as diagnostic stress indicators.

Keywords: beef; meat quality; glycogen; pre-slaughter stress

1. Introduction

Inducing stress in meat producing cattle from handling and transport is an unavoidable part of the pre-slaughter period [1-4]. The concept of stress has no universally agreed definition, but describes a state of threatened homeostasis brought on by nutritional, noxious or unpredictable stimuli (stressors) [5-9]. Stressors can be divided into nutritional, physical or psychological. Physical stimuli include pain, injury, exercise, temperature extremes and disturbances to the internal environment such as hypoglycaemia [7]. Psychological stressors include fear and anxiety [7]. After perceiving a threat, the central nervous system activates a biological defence of behavioural, autonomic nervous system and neuroendocrine responses to attempt to re-establish homeostasis [9]. The degree of stress experienced and homeostatic response will depend on the stressor type, intensity and duration [2, 10]. Furthermore, the way individual cattle perceive threats can vary widely depending on prior exposure and conditioning [9]. Changes in blood composition (electrolytes, metabolites and enzymes) can be used as physiological indicators to assess the response of the animal to pre-slaughter stress, transport and physical exertion. Adrenergic and glucocorticoid stimulation of lipolysis, glycogenolysis and gluconeogenesis results in increases in plasma glucose, lactate, and non-esterified fatty acids (NEFA). Water deprivation can be indicated by increases of electrolytes sodium and chloride. Pre-slaughter feed deprivation will further increase NEFA as well as β-hydroxybutyrate (βHB) [11]. Lipolysis and liberation of excess free fatty acids can reduce plasma magnesium levels (hypomagnesaemia) by binding and producing complexes in the blood [12]. The relationship of magnesium with the nervous system is cyclical in nature. When plasma magnesium is adequate it will dampen the stress response by reducing hypothalamic-pituitary-adrenal (HPA) axis activity and catecholamine release [12, 13]. However, hypomagnesaemia can induce a type of stress, of which the resulting catecholamine release further exacerbates low plasma magnesium by shifting magnesium into the intracellular fluid. Vigorous exercise or physical damage to the musculature results in a release of muscle enzyme indicators creatinine kinase (CK) and aspartate transaminase (AST).

Acute stress can be defined as lasting minutes to hours whilst chronic stress is days, months or years. The first stage of stress, synonymous to Cannon's "fight or flight" response, activates the sympatho-adrenalmedullary (SAM) system which releases catecholamines, notably adrenaline and noradrenaline, from the adrenal medulla [9, 14]. Catecholamines promote fuel mobilisation via glycogenolysis and lipolysis [9, 15]. Typically classified as catabolic, the effect of catecholamines on muscle protein metabolism is time-dependent and can have anabolic effects by suppressing protein degradation [16]. Activation of the second stress response, the HPA axis, simulates the synthesis and release of glucocorticoids from the adrenal cortex [9]. The HPA axis has a complimentary action to the sympathetic system. Circulating glucocorticoids, notably cortisol, promote energy mobilisation and amplify catecholamine-mediated responses to ensure glucose precursors, which along with depressing protein synthesis, can rapidly accelerate muscle glycogenolysis and muscle glycogen depletion [5, 9].

Depleted muscle glycogen reserves at slaughter increase the risk of curtailing post-mortem muscle acidification through inadequate precursors for anaerobic respiration and lactate production [1], and thus there is a need to develop strategies to mitigate the effects of glycogen depletion. Repletion rates of muscle glycogen in ruminants depend on muscle type, crude protein and metabolisable energy intake [17-19]. Dietary supplementation studies in cattle to improve muscle glycogen and meat quality to date have focused on high-energy concentrate rations [20, 21]. Knee et al [20] demonstrated that pasture fed cattle supplemented with a triticale-based high energy concentrate ration (12% crude protein, 12 MJ/kg DM energy) improved muscle glycogen after 1-2 weeks of feeding. Knee et al [20] surmised that liveweight gain could be used as an indicator to predict muscle glycogen repletion and estimated that cattle should be growing somewhere between 0.5 – 1.5 kg/head/day before slaughter to have adequate muscle glycogen [22]. To comply with market specifications concentrate supplements are unable to be fed to pasture assured cattle, thus studies investigating glycogen repletion of cattle on pasture are required.

A certain level of pre-slaughter stress is inevitable and Meat Standards Australia (MSA) guidelines and pathways have been very effective at improving pre-slaughter handling and animal welfare [23].

However if the pathway to slaughter is unknown or there is a mistake in the system, there is currently no objective mechanism of detecting before slaughter which cattle are at increased risk of producing poor quality meat. Further, the practice of providing a pre-slaughter rest period for glycogen repletion has not been verified, particularly in relation to 100% pasture raised cattle. Studies investigating rest periods have focused on periods of less than 24 hours duration [24-26].

The overall aims of this experiment were firstly; can blood metabolites distinguish between various levels of commercial pre-slaughter stress? We hypothesised that cattle subjected to greater quantities of stress pre-slaughter (simulated via mixing and transport variations) will have increased physiological markers of acute stress, muscle damage, feed deprivation, dehydration, positive acute phase proteins and a decrease of plasma magnesium that will associate with muscle glycogen and ultimate pH (pHu).

Secondly; if cattle were subjected to stress in the pre-slaughter period, will a two week rest period be beneficial for muscle glycogen repletion? We hypothesised that a rest period after transport and mixing stress will improve muscle glycogen at slaughter.

2. Materials and Methods

Experiments were approved and monitored by the animal ethics committee at Murdoch University (Perth, Western Australia) with permit number R2839/16. Experimental design and protocols were established by MSA pathways committee and aimed to mimic Australian commercial conditions with the aim to provide industry relevant recommendations.

The experiments utilised a total of 488 cattle in a replicated design with two simulated stress treatments (Treatment 1 mixing stress and Treatment 2 transport stress) and a slaughter interval comparison (Treatment 3 direct slaughter from transport versus a two week rest prior to slaughter). Experiment One had unmixed and mixed unfamiliar cattle while subjecting them to sea and road transport. Experiment Two had unmixed and mixed unfamiliar cattle and put them through commercial saleyards.

The cattle were all pasture raised *Bos taurus* (Hereford, Angus & Murray Grey), less than 24 months of age and had not been administered prophylactic antibiotics or growth promoting hormonal implants. Two geographical locations in Australia were used, King Island, Tasmania in Experiment One and northwestern mainland Tasmania in Experiment Two. Cattle were sourced from four properties in each location.

2.1. Cattle design

The two experiments tested three experimental treatments. Treatment 1 (mixing) consisted of five different groups; 1) never mixed steers (NMS) 2) never mixed heifers (NMH) 3) mixed steers (MS) 4) mixed heifers (MH) and 5) mixed steers and heifers (MSex). Treatment 2 (transport) consisted of sea and truck transport in Experiment One and a saleyard and truck transport in Experiment Two. Treatment 3 (slaughter interval) consisted of two different groups, direct slaughter or a two week rest on pasture immediately prior to slaughter.

Experimental groups were allocated on farm and balanced across mixing, transport and resting treatment to ensure no confounding in either experiment. These groups and cattle numbers within each of them are shown for Experiment One in Table 1, and Experiment Two in Table 2. The superscripts demonstrate common groups during transport and lairage.

Twenty-one days prior to transport, cattle were weighed, and those meeting expected market weight specifications were randomly allocated to the transport subgroups identified in Table 1 or Table 2, then returned to the paddock as a mob.

Mixing and transport treatments commenced on day twenty or twenty-one. Starting at 0600 AEST cattle on all four properties were simultaneously mustered, yarded, weighed and drafted into their previously allocated transport subgroups. Commercial double-story stock trailers collected the cattle. Each trailer contained seven stock pens that could hold six cattle per pen.

The processing plant used was the same for both experiments. Upon arrival cattle were held in their subgroups and were either slaughtered immediately or sent to pasture paddocks next to the lairage area for 14days, after which they were slaughtered.

The differences between the transport design for Experiment One is detailed in section 2.2 and for Experiment Two in section 2.3.

2.2. Experiment One transport methodology

Experiment One consisted of sea and road transport with the allocation of cattle numbers by farm and treatment detailed in Table 1, and a timeline of the methodology is depicted in Figure 1.

On day twenty-one cattle loading began at 1000 and finished at 1400 AEST. Trucking distance from farm gate to port ranged from 20 to 65 km.

Cattle allocated to Ship-A were distributed across the two trailers (orange and green) such that each trailer was a replicate containing, 2 pens each of NMS and NMH and 1 pen each of MS, MH and MSex (Table 1). The stock trailers were loaded directly into the hold of Ship-A alongside other commercial cattle stock trailers. Shipping time to Devonport wharf, Tasmania was 10 hours, docking at 0630 AEST the following morning, where stock trucks drove 131 km over two hours to the processing plant. On arrival all cattle from Ship-A went to direct slaughter (Kill 1).

Cattle allocated to Ship-B were collected by blue trucks and transported to the same ship-docking wharf. Here they were unloaded into yards at the wharf and drafted into allocated stock pens on the deck of the ship. Treatment group position on Ship-B was randomised using a Latin square to ensure no experimental bias due to the location on ship. Ship B travelled from King Island to Stanley wharf, docking at 0630 AEST the following morning (shipping time 13 hours). Cattle were unloaded from the ship at 0700 onto trucks and transported 22 km for 30minutes to the same processing plant. Upon arrival half of Ship-B cattle went for direct slaughter (Kill 1) and half went for 14 day paddock rest prior to slaughter (Kill 2).

The time off feed from muster to slaughter for Kill 1 was approximately 28 hours.

Table 1. Experiment One. Number of cattle by location, farm, mixing, transport and slaughter interval (Total slaughter count. Excludes those eliminated from the trial). Superscripts demonstrate common groups during transport and lairage.

		Not Rested (Kill 1)						Rested 14days (Kill 2)						Total Head
		NMS	NMH	MS	MH	MSex	Total	NMS	NMH	MS	MH	MSex	Total	
Source	Ship													
Farm 1	A Orange truck	6[a]		3[m]		2[s]	11						0	21
	A Green truck	6[b]		3[n]		1[t]	10						0	
	B Blue truck	8[c]		8[o]		3[u]	19	8[c]		8[v]		4[x]	20	39
Farm 2	A Orange truck	6[d]		3[m]		1[s]	10						0	21
	A Green truck	6[e]		3[n]		2[t]	11						0	
	B Blue truck	8[f]		8[o]		4[u]	20	8[f]		8[v]		4[x]	20	40
Farm 3	A Orange truck		6[g]		3[p]	2[s]	11						0	21
	A Green truck		6[h]		3[q]	1[t]	10							
	B Blue truck		8[i]		8[r]	4[u]	20		8[i]		8[w]	4[x]	20	40
Farm 4	A Orange truck		6[j]		3[p]	1[s]	10							21
	A Green truck		6[k]		3[q]	2[t]	11							
	B Blue truck		8[l]		8[r]	4[u]	20		8[l]		8[w]	4[x]	20	40
	Total Ship-A	24	24	12	12	12	84	0	0	0	0	0	0	84
	Total Ship-B	16	16	16	16	15	79	16	16	16	16	16	80	159
	Total King Island	40	40	28	28	27	163	16	16	16	16	16	80	243
	Time from point of muster to slaughter	28 hours						1 hour						

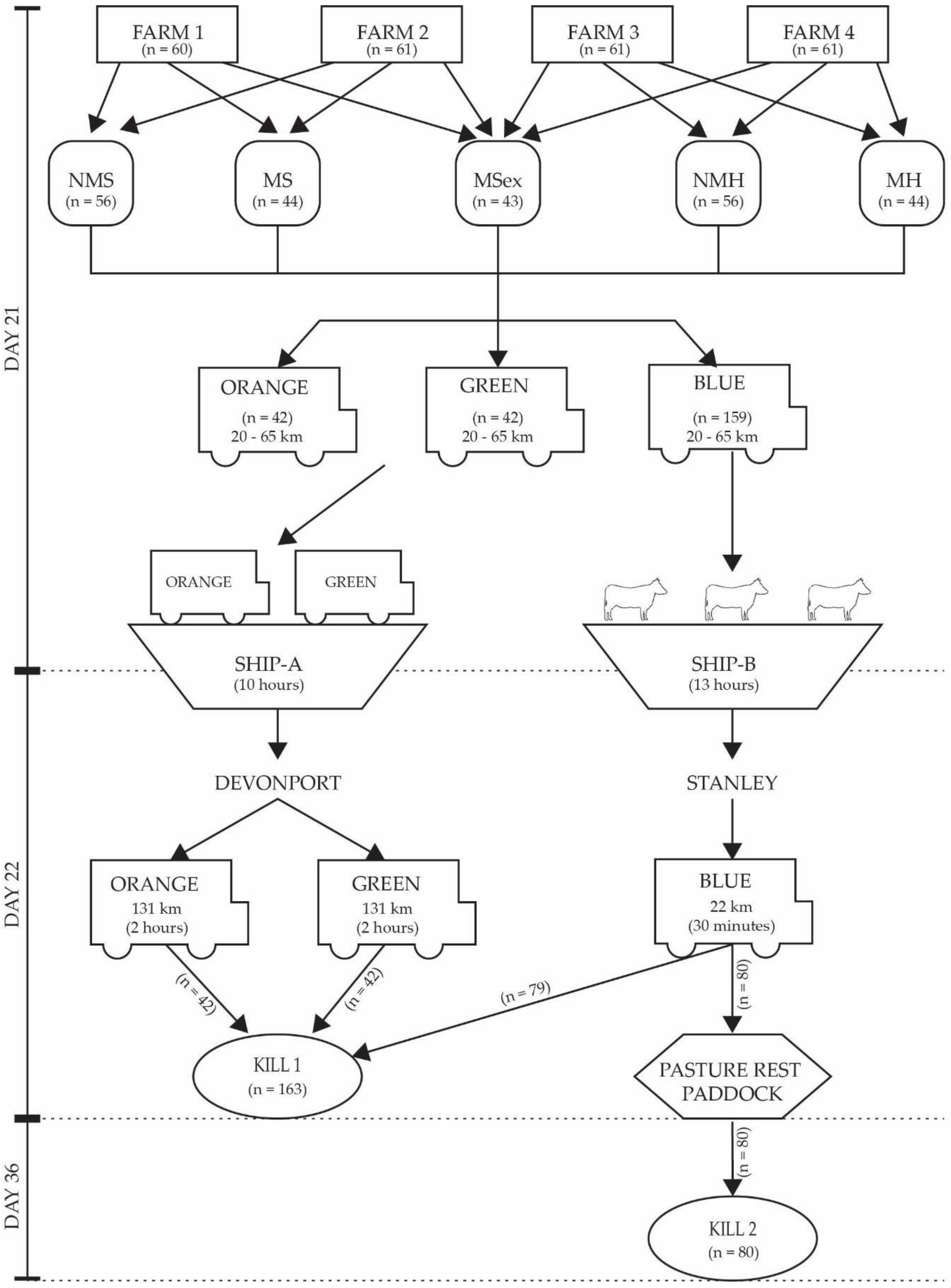

Figure 1 Experiment One diagram of timeline methodology for mixing, transport and slaughter interval. NMS, never mixed steers; NMH, never mixed heifers; MS, mixed steers; MH, mixed heifers; MSex, mixed steers and heifers. A truck symbol denotes trucking pathway and cattle symbol denotes cattle were unloaded and on foot rather than in trucks. Arrows represent direction of cattle movement and dotted lines represent timeline. Any parameter that intersects the dotted line has occurred over multiple days.

2.3. Experiment Two

Experiment Two transport protocol was a saleyard pathway where cattle were put through a simulated auction day to represent commercial Australian saleyards. Transport treatment consisted of 3 groups; Control (cattle transported directly from farm to processing plant), Saleyard-A and Saleyard-B. Table 2 details the allocation numbers by farm and treatment and Figure 2 details the timeline methodology for Experiment Two.

On day twenty, cattle loading began at 1200 and finished at 1600 AEST. Never mixed steer and heifer groups remained on farm. All trucks drove first to Saleyard-A, arriving between 1700 – 1900 AEST. Distance travelled from farm gate to Saleyard-A ranged from 37 – 194 km. The allocated cattle for Saleyard-A were unloaded into pens which corresponded with their subgroup (Table 2). The remaining 18 cattle on the stock trailer were transported a further 117 km to Saleyard-B, arriving between 2000 – 2200 AEST where again cattle were unloaded into pens corresponding with their subgroup.

Day 21 (Saleyard-A) and Day 22 (Saleyard-B) were saleyard simulations. Cattle underwent handling as per a normal commercial sale which included weighing, paint branding, moving through sale pens and a mock auction. When not under sale conditions, cattle were held in adjacent dirt pens supplemented with hay and silage.

After saleyard simulations had finished on day 22, all saleyard cattle were transported to the processing plant (243 km for Saleyard-B, 134 km for Saleyard-A). On arrival, half of the cattle were kept overnight in lairage before slaughter the following day (day 23, Kill 3). Time from muster to point of slaughter for Kill 3 cattle was approximately 76 hours. The remaining cattle were paddock rested in their treatment groups for two weeks prior to slaughter (Day 37, Kill 4).

The never mixed cattle for direct slaughter were transported from farmgate to processing plant on Day 22 kept in lairage pens overnight. Day 23 direct slaughter cattle (n = 120) were processed (Kill 3). On day 35 the remaining never mixed cattle were transported from farmgate to the processing plant and held in lairage pens overnight. Day 36 was the final processing day for all remaining rested cattle (Kill 4, n = 117).

Table 2. Experiment Two. Number of cattle by location, farm, mixing, transport and slaughter interval (Total slaughter count. Excludes three cattle eliminated from the trial). Superscripts demonstrate common groups during transport and lairage.

		Not Rested						Rested 14days						Total Head
		NMS	NMH	MS	MH	MSex	Total	NMS	NMH	MS	MH	MSex	Total	
Source	Direct/Saleyard													
Farm 5	Direct	6[a]<br>6[b]					12	6[w]<br>6[x]					12	24
	Saleyard-A			3[i]<br>3[j]		2[q]<br>1[r]	9			2[ee]<br>3[ff]		1[nn]<br>2[oo]	8	17
	Saleyard-B			3[k]<br>3[l]		2[s]<br>1[t]	9			3[hh]<br>3[ii]		1[pp]<br>2[qq]	9	18
Farm 6	Direct	6[c]<br>6[d]					12	6[y]<br>6[z]					12	24
	Saleyard-A			3[i]<br>3[j]		1[q]<br>2[r]	9			3[ee]<br>2[ff]		2[nn]<br>1[oo]	8	17
	Saleyard-B			3[k]<br>3[l]		1[s]<br>2[t]	9			3[hh]<br>3[ii]		2[pp]<br>1[qq]	9	18
Farm 7	Direct		6[e]<br>6[f]				12		6[aa]<br>6[bb]				12	24
	Saleyard-A				3[m]<br>3[n]	2[q]<br>1[r]	9				3[jj]<br>3[kk]	1[nn]<br>2[oo]	9	18
	Saleyard-B				3[o]<br>3[p]	2[s]<br>1[t]	9				3[ll]<br>3[mm]	1[pp]<br>2[qq]	9	18
Farm 8	Direct		6[g]<br>6[h]				12		6[cc]<br>6[dd]				12	24
	Saleyard-A				3[m]<br>3[n]	1[q]<br>2[r]	9				3[jj]<br>3[kk]	2[nn]<br>1[oo]	9	18
	Saleyard-B				3[o]<br>3[p]	1[s]<br>2[t]	9				3[ll]<br>2[mm]	2[pp]<br>1[qq]	8	17
	Total Direct	24	24				48	24	24				48	96
	Total Saleyard-A			12	12	12	36			10	12	12	34	70
	Total Saleyard-B			12	12	12	36			12	11	12	35	71
	Grand Total	24	24	24	24	24	120	24	24	22	23	24	117	237
	Time from point of muster to slaughter	76 hours						1 hour						

animals MDPI

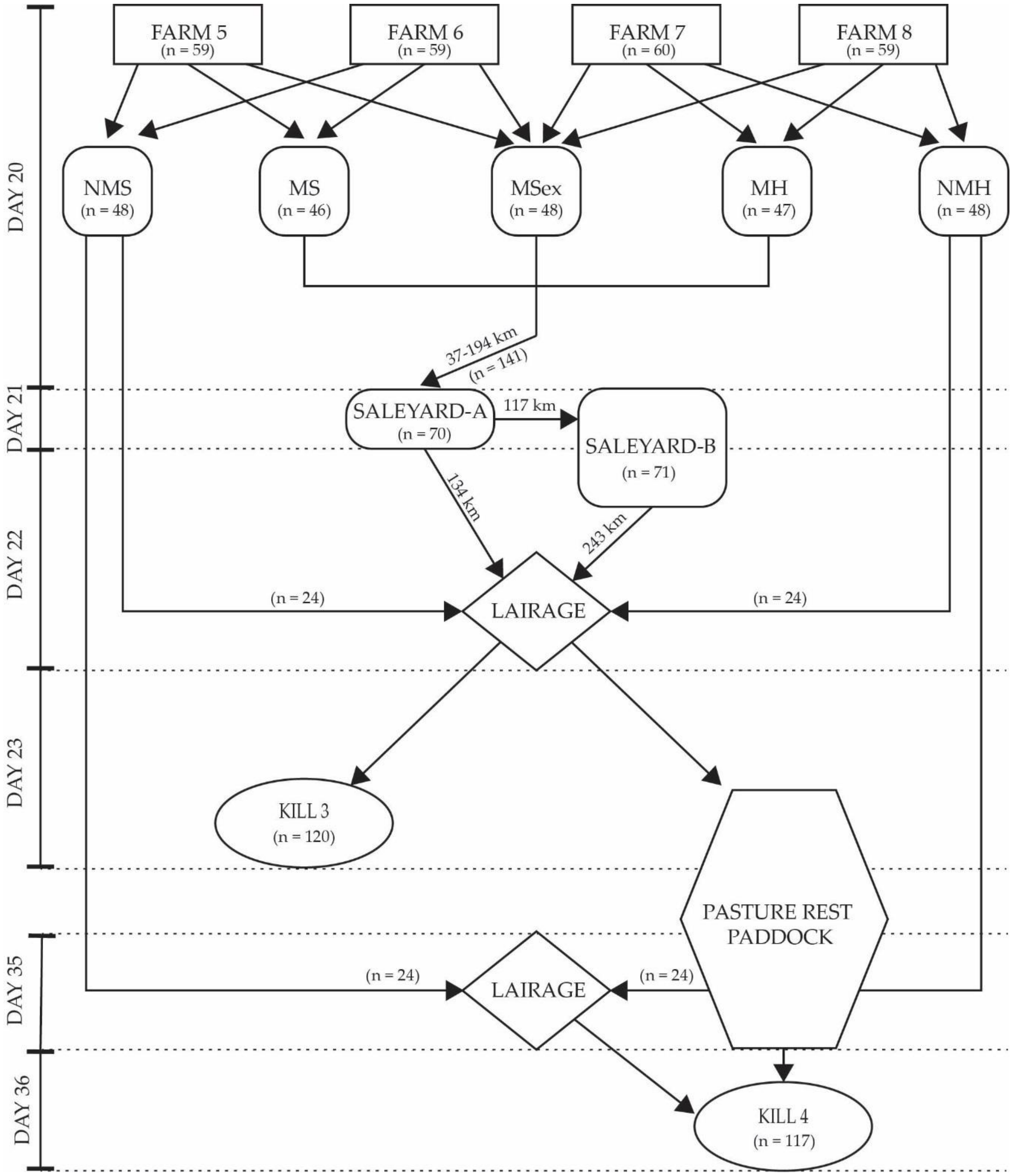

Figure 2 Experiment Two diagram of timeline methodology for mixing, transport and slaughter interval. NMS, never mixed steers; NMH, never mixed heifers; MS, mixed steers; MH, mixed heifers; MSex, mixed steers and heifers. Arrows represent direction of cattle movement and dotted lines represent timeline. Any parameter that intersects the dotted line has occurred over multiple days.

2.4. Slaughter and carcass measurements

The day following slaughter, all carcasses were graded by qualified Meat Standards Australia and AUS-MEAT graders (MLA, 2006). Carcasses were identified with a carcass ticket and measurements taken using the methodology described in Loudon et al. [27]. Measurements included Eye Muscle Area (EMA), Ossification, Meat colour, Marbling, rump fat depth (P8), Rib fat depth, Ultimate pH and loin temperature. For a carcass to be eligible for MSA compliance they must have rib fat ≥3 mm and ultimate pH ≤5.7.

2.5. Blood sampling and plasma analysis

Blood was collected from each animal during exsanguination into 1x 9mL lithium heparin Vacuette® tubes (Greiner bio-one, Austria). The lithium heparin tubes were inverted gently seven times. Tubes were kept in shaved ice in a cooler box until centrifugation at 2000RPM for 15 minutes within 2-4 hours of collection. Plasma was transferred into 3x 2mL tubes and frozen at -20 °C until further analysis. All samples were analysed within one month of collection.

The plasma aliquot was analysed for glucose, lactate, NEFA, magnesium (Mg), sodium (Na), chloride (Cl) concentrations and CK and AST activity. Assays were performed via Beckman kits designed for the Olympus AU400 Automated Chemistry Analyser (Olympus Optical CO. Ltd, Melville, NY). Reagent kits were used for glucose (OSR6121), lactate (OSR1693), magnesium (OSR6189), CK (OSR6279) and AST (OSR6209). NEFA analysis was conducted using a separate kit (C Kit Wako Pure Chemical Ind., Osaka, Japan; modified for the Olympus AU400 Automated Chemistry Analyser). Sodium and chloride analyses were conducted using the Roche Cobas Integra 400plus with reagent a reagent kit for electrolytes (0738085). All analyses were run according to the kit protocols with calibration and quality control adhered to.

The second plasma aliquot was analysed for βHB, haptoglobin (HP-T) and ceruloplasmin (Cp) analysis as a batch sample by enzymatic methods using Olympus AU400 Clinical Chemistry Analyser (Olympus Optical CO. Ltd, Melville, NY). A reagent kit was used for βHB (Randox Ranbut Reagent kit, RB 1007) and in-house methods were used for HP-T (NTM-62 as per Eckersall et al., 1999) and Cp (NTR-23 as per Siotto et al., 2014).

2.6. Muscle Collection and Glycogen analysis

A small core sample (approximately 10gram) of the *m. longissiumus thoracis* (loin) was taken at approximately 40 minutes after death. A 10cm x 1.5cm stainless steel drill bit on a hand-held electric drill was used to take the sample quartering site. All fat was removed before placing in a 5mL plastic screw top tube, labelled with the corresponding body number. Samples were placed immediately on ice then stored at -20°C and shipped frozen for later analysis of glycogen concentration.

Muscle samples were assayed for glycogen, free glucose and lactate. The sum of these were calculated to determine total muscle glycogen to account for degradation of glycogen between exsanguination and sampling. Muscle glycogen concentration was determined by the enzymatic method of Chan and Exton [28] excluding the filter paper step. The assay was performed on an Olympus AU 400 auto analyser (Olympus Diagnostics, Tokyo, Japan). Lactate concentration in muscle was analysed on the glycogen homogenates by the method described by Noll [29].

2.7. Statistical Analysis

Several blood measurements exhibited a high degree of heterogeneity of variance and so natural logarithm transformations were applied to CK, AST, HP-T and CP. For CK, AST, HP-T and CP all subsequent analyses were undertaken using the transformed data.

All statistical analyses were performed in R [30]. Correlation and principle analysis was performed on the combined data of Experiment One and Two. Correlation analysis was performed using Pearson correlation coefficients and where missing data was encountered correlations were estimated on a pairwise complete basis, i.e. the set of complete cases were analysed. Principal component analysis was used to identify underlying latent structures among traits in the multivariate data set. The figures were created using the ggplot2 R package [31].

Muscle and plasma metabolites were analysed using linear mixed effects models via the lme4 R package [32]. Experiment One and Experiment Two were analysed independently, with mixing group, transport type (boat or saleyard), and slaughter interval (direct or rested) used as fixed effects, and property of origin used as a random term. In this case only p-values for the significance of these terms are reported.

3. Results

3.1. Descriptive Statistics

Table 3 shows the descriptive statistics for the MSA carcass grading characteristics separated into Experiment One and Experiment Two cohorts. The mean carcass weights were similar between the two locations however the range was larger in Experiment Two (188.2 – 425.8 kg) compared to Experiment One (206.8 – 322.0 kg) though the maximum ossification score was the same for both cohorts.

Table 3. Summary of MSA grading characteristics by experiment.

Variable	Experiment One (n=243)				Experiment Two (n=240)			
	Mean	SD	Min	Max	Mean	SD	Min	Max
Carcass weight (kg)	270.7	20.8	206.8	322.0	265.3	55.3	188.2	425.8
P8 (mm)	7.7	2.0	5.0	15.0	7.5	2.7	2.0	20.0
EMA (cm^2)	70.0	8.3	50.0	96.0	68.7	10.1	50.0	140.0
Ribfat (mm)	6.5	2.6	2.0	14.0	5.7	3.3	1.0	21.0
Ossification score	166.7	26.4	120.0	230.0	157.8	25.5	100.0	230.0
MSA Marble Score	345.6	67.0	170.0	740.0	300.5	70.1	130.0	520.0

Table 4 shows the MSA non-compliance for ultimate pH and ribfat for each group. There was a stark difference in ultimate pH in Experiment One, with 39.3% classified as a dark cutter in the direct slaughter group compared to 3.6% in the rested cattle. Experiment Two had the opposite response where dark cutting was higher in the rested cohort at 21.7% compared to 16.7% in Kill 3.

Table 4. Summary of MSA non-compliance for pHu and ribfat by location and kill.

Location	Slaughter Group	pH >5.7	Ribfat <3mm
**Experiment One (direct slaughter)**	Kill 1 (n = 163)	64	3
**Experiment One (rested)**	Kill 2 (n = 80)	3	4
**Experiment Two (direct slaughter)**	Kill 3 (n = 120)	20	14
**Experiment Two (rested)**	Kill 4 (n = 120)	26	18

Table 5 shows the raw data means, standard deviation, minimum and maximum values for plasma and muscle indicators. HP-T, Cp, CK and AST activities had a skewed distribution and were log-transformed to normalise the data. The biochemical means were similar between the two experiments with the notable exception of plasma CK activities which were approximately twice as high in Experiment One versus Experiment Two. Across the biomarkers there was a marked elevation in plasma glucose and L-lactate concentrations and CK activity. Positive acute phase proteins plasma HP-T and Cp concentrations were mildly elevated whilst the means for βHB, NEFA, Mg and AST activity appeared to be within normal reference ranges for cattle. The elevation and range of plasma Na and Cl concentrations suggests that some cattle were experiencing dehydration.

Table 5. Descriptive statistics of plasma metabolites, enzymes and protein biomarkers taken immediately at slaughter and M. longissimus lumborum glycogen and lactate.

**Location.**	Variable	Mean	SD	Min	Max	Published normal basal concentrations
Experiment One	Glucose (mmol/L)	7.42	1.22	3.69	14.51	1.9 – 3.8[a]
Experiment Two		6.23	0.89	4.66	10.34	
Experiment One	Lactate (mmol/L)	14.48	3.30	7.15	23.48	0.6 - 2.2[a]
Experiment Two		12.01	2.49	5.78	21.26	
Experiment One	NEFA (mmol/L)	0.35	0.17	0.08	0.98	<0.4[a]
Experiment Two		0.58	0.27	0.11	1.52	
Experiment One	βHB (mmol/L)	0.21	0.13	0.04	0.68	0.35 – 0.47[a]
Experiment Two		0.31	0.14	0.03	0.85	
Experiment One	Magnesium (mmol/L)	0.79	0.10	0.52	1.10	0.74 – 1.10[a]
Experiment Two		0.78	0.11	0.48	1.05	
Experiment One	CK (IU/L)	1137.86	1346.48	113.30	9384.90	35 – 280[a]
Experiment Two		596.79	509.82	144.4	4791.00	
Experiment One	AST (IU/L)	113.28	71.14	58.36	636.17	78 – 132[a]
Experiment Two		110.66	48.58	55.57	440.57	
Experiment One	Sodium (mmol/L)	147.72	3.39	125.70	160.80	132 – 152[a]
Experiment Two		147.99	3.51	128.40	155.80	
Experiment One	Chloride (mmol/L)	97.85	2.86	91.50	113.60	95 – 110[a]
Experiment Two		98.07	3.74	91.40	119.50	
Experiment One	Haptoglobin (mg/mL)	0.35	0.30	0.03	1.80	0.0 – 0.2[b]
Experiment Two		0.26	0.49	0.01	3.24	
Experiment One	Ceruloplasmin (IU/L)	112.02	22.17	50.00	202.00	15 – 68[c]
Experiment Two		79.96	40.34	20.00	258.00	
Experiment One	Muscle Glycogen (g/100g)	0.95	0.20	0.40	1.56	
Experiment Two		1.18	0.22	0.31	1.78	

[a] [33], [b] [34, 35], [c] [36]

The underlying latent structure of the plasma and muscle metabolite data was explored using a correlation matrix and principal component analysis (PCA) (Figure 3). For PCA, the closer variables are in the same plane, the higher the relationship. For example, the loadings for NEFA and βHB concentrations are pointing in the same direction which indicates that they are highly correlated, and similarly for AST and CK activity as well as Cp, L-lactate and glucose concentrations. Variables in opposite planes indicate negative correlation. The first two principal components accounted for approximately 42% of the variance (Figure 3).

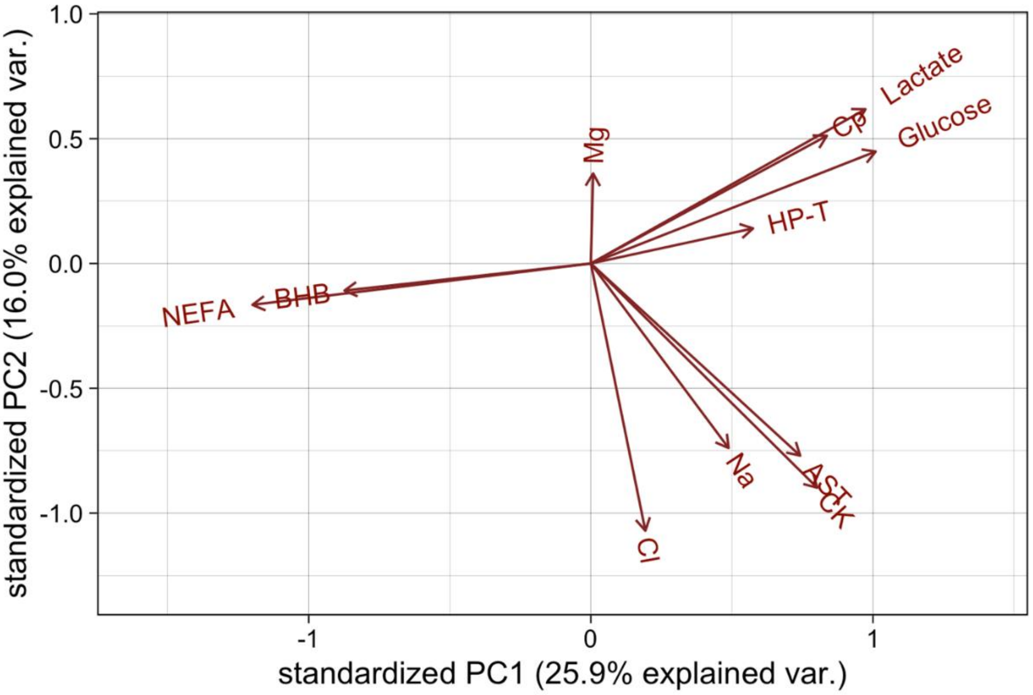

Figure 3. Loadings of each variable on the first and second principal components.

The majority of the correlations (Table 6) were statistically significant (P <0.05), partly as a function of sample size, even when correlations were low. To ensure practical relevance only correlations with P<0.05 and $r^2 > 0.1$ are emphasised (Table 6 "bolded").

Table 6. Correlation coefficients for blood and muscle metabolites and ultimate pH. Bolded correlations indicate significance at P <0.05 and $r^2 > 0.1$

	Glucose	Lactate	NEFA	Mg	βHB	Cl	Na	HP-T (log)	CK (log)	AST (log)	Cp (log)	Glycogen	pHu
Glucose	1.00	**0.50**	**-0.47**	0.04	-0.21	-0.02	-0.01	0.18	0.20	0.15	**0.32**	**-0.34**	0.13
Lactate		1.00	**-0.46**	0.11	-0.24	-0.22	0.27	0.21	0.01	0.06	0.29	-0.17	0.02
NEFA			1.00	0.03	**0.42**	-0.09	-0.17	-0.25	-0.24	-0.19	**-0.38**	0.20	-0.07
Mg				1.00	0.08	0.00	0.01	-0.11	-0.07	-0.09	0.15	0.02	-0.07
BHB					1.00	-0.02	-0.07	-0.12	-0.19	-0.18	-0.31	0.22	-0.15
Cl						1.00	**0.50**	0.00	0.25	0.09	-0.03	-0.11	0.07
Na							1.00	0.08	0.11	0.03	-0.08	0.04	-0.02
HP-T (log)								1.00	0.05	0.13	0.17	-0.09	-0.04
CK (log)									1.00	**0.70**	0.14	**-0.52**	**0.42**
AST (log)										1.00	0.11	**-0.40**	**0.36**
Cp (log)											1.00	-0.22	0.18
Glycogen												1.00	**-0.54**

Markers responding to acute stress glucose & lactate were positively correlated (P <0.01, $r^2 = 0.25$) as were markers of tissue mobilisation βHB and NEFA (P <0.01, $r^2 = 0.18$). Blood NEFA was negatively correlated to both glucose (P <0.01, $r^2 = 0.22$) and lactate (P <0.01, $r^2 = 0.21$), however βHB was not correlated with glucose or lactate.

Markers of dehydration sodium and chloride were positively correlated (P <0.01, $r^2 = 0.25$), however these were not correlated with muscle glycogen or ultimate pH.

Plasma CK and AST had the strongest correlation of all the indicators (P <0.01, $r^2 = 0.49$) and were positively correlated with ultimate pH (P<0.01, $r^2 = 0.18$) and (P<0.01, $r^2 = 0.13$) respectively indicating cattle with higher muscle activity/trauma had higher rate of glycogen depletion pre-slaughter.

Plasma Cp concentrations had a weak positive correlation with glucose (P<0.01, $r^2 = 0.1$) and a weak negative correlation with NEFA (P<0.01, $r^2 = 0.14$). Cp is a positive acute phase protein as well as an indicator of copper status. As the other positive acute phase protein analysed HP-T had no significant correlations the practical significance of this marker is questionable.

When blood variable correlations were broken into the two separate locations, NEFA had a different pattern in the Experiment One cohort compared with Experiment Two cattle (Figure 4). NEFA was positively correlated with βHB in Experiment Two but there was no correlation in Experiment One.

In Experiment One, the NEFA response appeared to be blunted with a mean and range significantly lower than the Experiment Two cohort. Experiment One had a lower NEFA mean (0.35 ± 0.17 mmol/L) and a smaller range (0.08 – 0.98 mmol/L) compared to Experiment Two mean (0.58 ± 0.27 mmol/L) and range (0.11 – 1.52 mmol/L). NEFA had a negative curved-linear correlation with glucose in both experiments and a negative linear correlation with lactate.

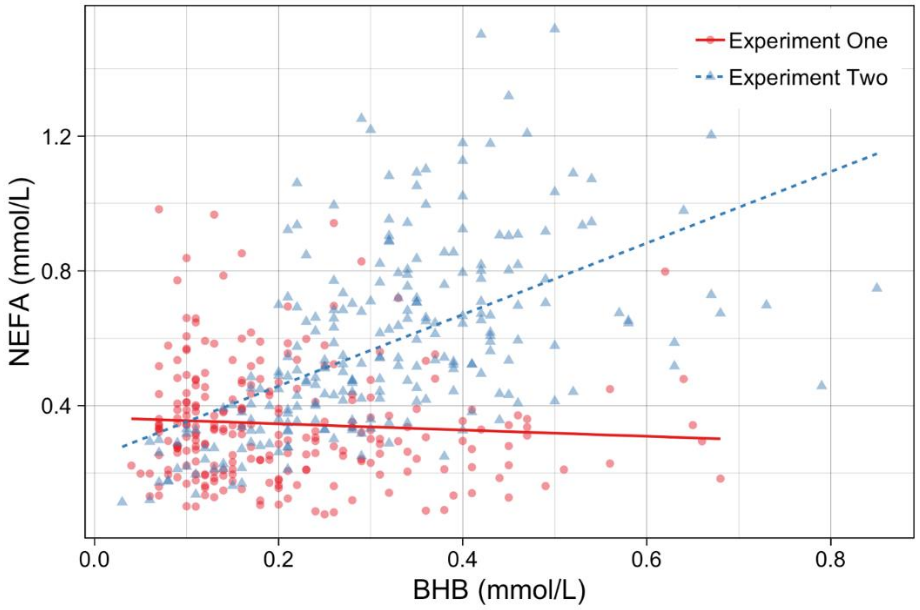

Figure 4. The relationship between NEFA & βHB. Red dots represent Experiment One cattle. Blue triangles represent Experiment Two cattle. The lines are fitted linear regression lines for the two groups.

As expected, muscle glycogen content was negatively correlated with plasma glucose concentrations (P<0.01, $r^2 = 0.12$), plasma CK (P<0.01, $r^2 = 0.27$) and AST activity (P<0.01, $r^2 = 0.16$) and muscle pHu (P<0.01, $r^2 = 0.29$), findings consistent with increased glycogenolysis resulting from acute stress and muscular activity/trauma resulting in a higher ultimate pHu.

3.2. Treatment One: Mixing

3.2.1. Experiment One

Mixing of unfamiliar cattle had minimal effect on physiological variables compared to unmixed cohorts as indicated by limited significance of findings (Table 7). However, there was a significant difference in Mg concentrations and AST activity (P <0.01) for mixing groups, with plasma magnesium concentrations differing (P <0.01) between NMS and MS. The NMS cattle exhibited higher plasma Mg concentrations than MS cattle (0.85 ± 0.03 versus 0.78 ± 0.03 mmol/L).

Plasma AST activity was highest and significantly different in the MH groups at 146.02 ± 10.10 IU/L compared to other groups. There was no significant difference for the other groups.

The overall effect of mixing on plasma CK activity was not significant (P >0.1) but the pattern of estimated means for the mixing groups was similar to plasma AST activity.

3.2.2. Experiment Two

For Experiment Two, mixing (treatment 1) was not fitted into the statistical model as it is confounded with treatment 3 (saleyard or direct consignment). The control cattle were never mixed heifers or steers and were consigned directly to slaughter, whereas the cattle that went through a saleyard pathway were all mixed.

3.3. Treatment Two: Transport Method

3.3.1. Experiment One

For Experiment One, cattle travelling on the Ship-A had significantly higher (P <0.01) βHB and HP-T and chloride concentrations (P <0.01 Table 7). The βHB concentration for Ship-A transport was 0.26 ± 0.02 mmol/L and 0.19 ± 0.01 mmol/L for Ship-B. Plasma HP-T concentrations of cattle on Ship-A was 0.38 ± 0.05 mg/mL and 0.23 ± 0.02 mg/mL for Ship-B. Plasma chloride concentration of cattle on Ship-A was 97.98 ± 0.56 mmol/L compared to 97.0 ± 0.49 mmol/L for Ship-B. While the differences in plasma sodium concentrations between the ships were not significant (P = 0.09), they followed a similar pattern to plasma chloride concentrations with cattle travelling on Ship-A having higher estimated means, 148.49 ± 0.54 mmol/L than those on the Ship-B 147.55 ± 0.35 mmol/L.

3.3.2. Experiment Two

For Experiment Two cattle transported through Saleyard-A, plasma NEFA concentrations were 0.11 ± 0.04 mmol/L higher than the direct consignment groups (P <0.01). There was no significant difference between Saleyard-B and control for plasma NEFA concentrations. The plasma βHB concentration was 0.04 ± 0.02 mmol/L lower in Saleyard-B cattle compared to the control (P <0.01). There was no significant difference between other physiological indicators and transport pathways for Experiment Two.

3.4. Treatment Three: Slaughter interval

3.4.1. Experiment One

Resting at pasture 2wks prior to slaughter had a significant effect on circulating L-lactate and HP-T concentrations and CK and AST activity and muscle glycogen concentration (P <0.01 Table 7). The plasma L-lactate concentration in direct slaughter cattle was 13.4 ± 0.50 mmol/L verses 16.6 ± 0.68 mmol/L of rested cattle. The plasma CK and AST enzyme activities were both significantly higher in direct slaughter cattle in comparison to rested cohorts (P <0.05, Table 7). The plasma CK activity was 1128.55 ± 99.26 IU/L for direct consignment cattle versus 310.59 ± 37.85 IU/L for rested. The plasma AST activity for saleyard versus direct consignment was 134.16 ± 3.94 IU/L and 105.43 ± 5.88 IU/L, respectively. The plasma HP-T concentration was higher (P <0.01 Table 7) in rested cattle at 0.52 ± 0.08 mg/mL compared to direct slaughter at 0.17 ± 0.0 mg/mL. Slaughter interval had a significant impact on muscle glycogen concentration with those rested having a higher estimated means 1.13 ± 0.04 g/100g compared to 0.92 ± 0.01 g/100g for direct slaughter.

3.4.2. Experiment Two

In Experiment Two, a two week rest period prior to slaughter had a significant effect on the plasma CK activity andβHB and HP-T concentrations. The HP-T concentrations had the opposite response for slaughter interval to Experiment One where estimated means were lower in rested cattle at 0.09 ± 0.09 mg/mL compared to direct slaughter at 0.14 ± 0.14 mg/mL(P <0.01 Table 7).

The plasma βHB concentration was also lower in rested cattle, 0.27 ± 0.02 mmol/L vs 0.36 ± 0.02 mmol/L in direct consignment cattle. There was the same increased plasma CK concentration with direct slaughter however the magnitude of effect was significantly less than Experiment One, 537 ± 28.4 IU/L direct kill vs 417 ± 22.3 IU/L for rested cattle.

3.5. Carcass Characteristics

Sex was significantly associated with plasma βHB and NEFA concentrations in the cattle used in Experiment One (p<0.05). βHB concentrations were 0.13 ± 0.05 mmol/L higher for heifers and NEFA concentrations were 0.13 ± 0.06 mmol/L lower compared to steers.

Muscle glycogen concentration was 0.13 ± 0.06 mmol/L and 0.10 ± 0.04 mmol/L higher in heifers (P <0.05) for Experiment One and Experiment Two respectively.

Rib fat thickness was significantly associated with physiological indicators only in Experiment Two cattle (P <0.05). A 1 mm increase in rib fat was associated with an increase in plasma glucose concentration by 0.07 ± 0.03 mmol/L, Cp by 0.01 ± 0.004 IU/L and plasma magnesium concentration by 0.01 ± 0.003 mmol/L while plasma NEFA concentrations were 0.02 ± 0.01 mmol/L lower.

The P8 fat depth was significantly associated (P<0.05) with plasma NEFA and magnesium concentrations in Experiment One cattle. A 1 mm increase in P8 fat was associated with an increase in plasma NEFA concentrations of 0.02 ± 0.01 mmol/L and magnesium concentration of 0.012 ± 0.01 mmol/L. Marbling score was significantly associated (P <0.05) with muscle glycogen in Experiment One cattle with 100 point increase in MSA marbling score being associated with a 0.03 ± 0.002 g/100g decrease in total muscle glycogen content.

The HSCW was significantly associated plasma indicators in Experiment Two cattle only ($P < 0.01$). A 1 kg increase in carcass weight was associated with an increase of 0.02 ± 0.01 mmol/L of Na concentration and decreased Cp concentration of 0.001 ± 0.001 mmol/L.

Table 7 P values for the effects of mixing, transport, slaughter interval and pHu on plasma and muscle metabolites concentration. Bold represents P <0.05.

	L lactate		Glucose		CK		AST		βHB		NEFA		Mg		Na		Cl		HP-T (log)		Cp		Glycogen	
Experiment #	1	2	1	2	1	2	1	2	1	2	1	2	1	2	1	2	1	2	1	2	1	2	1	2
**Treatment 1 (Mixing)**	0.706		0.394		0.112		**0.012**		0.222		0.168		**0.005**		0.771		0.820		0.506		0.977		0.606	
**Treatment 2 (Transport Method)**	0.889	0.462	0.987	0.139	0.629	0.266	0.879	0.397	**0.001**	**0.010**	0.259	**0.002**	0.394	0.263	0.093	0.107	**0.005**	0.697	**0.001**	0.789	0.178	0.551	0.495	0.379
**Treatment 3 (Slaughter Interval)**	< **0.001**	0.565	0.852	0.170	< **0.001**	**0.002**	**0.001**	0.781	0.061	< **0.001**	0.233	0.627	0.211	0.145	0.235	0.754	< 0.001	0.601	< **0.001**	**0.007**	0.643	0.547	< **0.001**	0.305
**pHu**	0.120	0.488	0.886	0.316	< **0.001**	< **0.001**	< **0.001**	< **0.001**	0.417	0.737	0.615	0.453	0.273	0.317	0.671	0.749	0.539	0.777	0.944	0.419	0.153	0.607	< **0.001**	< **0.001**

4. Discussion

Contrary to the hypothesis, pre-slaughter physical or psychological stress, due to mixing of unfamiliar cattle, transportation type and selling method, did not have any consistent effects on plasma biomarkers or muscle ultimate pH or glycogen.

4.1. Correlations and principle component analysis of blood metabolites

While the principle component analysis and correlation matrix demonstrated certain expected correlations, the only plasma markers with significant correlations with muscle glycogen concentrations were glucose and the haematological markers of muscle damage, CK and AST. The muscle damage enzyme markers were the only biomarkers that correlated with muscle pHu. The lack of association with ultimate pH highlights the insensitivity of this measure, as muscle glycogen must deplete to levels below approximately 0.7 – 0.8 g/100g in the live animal before muscle pHu shows any change [37]. Muscle glycogen is a more sensitive indicator of an cattle susceptibility to cut dark.

Plasma glucose and L-lactate concentrations were highly correlated, and the very high concentrations detected in this experiment suggests there was a marked adrenergic surge driving muscle glycogenolysis and hepatic glycogenolysis in the experimental cattle. While plasma glucose was negatively correlated with muscle glycogen supporting the long established link between acute stress and slaughter glycogen concentrations [1], there was no association between muscle glycogen content and plasma L-lactate concentrations. This contrasts with a recent extended transport treatment in beef steers that found positive correlations between plasma glucose and L-lactate concentrations and muscle pHu [38]. In an analysis of pre and post transport behavioural scores, plasma metabolites and meat quality, Gruber et al. [39] found a low correlation of slaughter plasma glucose and L-lactate ($r = 0.16$) and interestingly L-lactate had a negative correlation with loin pH ($r = -0.30$), however all cattle evaluated were compliant for ultimate pH (pH <5.8). Variations in acute stress and pre-slaughter muscular activity will influence the circulating concentrations of these metabolites. Plasma L-lactate is derived during intense exercise and contraction linked muscle glycogenolysis or from acute adrenergic stress where a surge in glycolysis can occur at two to three times the rate of oxidative phosphorylation thus excess pyruvate is diverted to lactate [40, 41]. While the principle role of glycogen storage in muscle is to provide a readily mobilised form of energy for exercise, the rates of muscle contraction induced glycogen depletion are dependent on exercise intensity [18]. Prolonged exercise intensities above 60% VO2 max are associated muscle glycogen depletion whereas at low exercise intensities of around 30% VO2 max there is little shift in muscle glycogen concentrations [18, 42, 43]. Thus, the high circulating L-lactate concentrations in the present study are likely due to rapid peripheral production at slaughter rather than muscle glycogen depletion due to intense exercise.

There were no significant correlation between plasma NEFA and βHB concentrations with muscle glycogen or ultimate pH. The elevated range of circulating NEFA and βHB concentrations across both experiments suggests that certain cattle were mobilising fat for energy metabolism. While still within the reference range, the means for circulating NEFA and βHB concentrations were higher in Experiment Two suggesting greater lipid mobilisation in these cattle. The negative correlation between plasma NEFA and glucose concentrations was expected. Blood glucose declines with feed

deprivation in ruminants, and concomitant hydrolysis of fat stores for energy results in an increase in circulating NEFA. Oxidation of NEFA can have two fates in the liver; complete oxidation through the Krebs cycle or when available acetyl-CoA precursors overwhelm the cycle incomplete oxidation via hepatic ketogenesis occurs, resulting in an elevation of circulating βHB concentrations [44-46]. Adrenaline can cause a fourfold increase in lipolysis in ruminants hence an increase in circulating NEFA concentrations can occur from catecholamine driven lipolysis from acute stress or feed deprivation [11, 44]. There was no correlation between circulating βHB and glucose concentrations in either experiment suggesting that feed deprivation was not to the extent of causing severe energy imbalance. Interestingly, the correlations between βHB and plasma NEFA concentrations differed between the two experiments. The lack of correlation between circulating NEFA and βHB concentrations in Experiment One may simply represent a shorter period of feed deprivation in these cattle. The interval from muster to slaughter was approximately three times longer in Experiment Two, and even though hay and silage were offered, the frequent trucking, saleyard movement and unfamiliar environment may have reduced appetite in these cattle, therefore increasing fat metabolism. Alternatively, the lack of correlation between circulating NEFA and βHB concentrations in Experiment One may be due to a blunting of adrenergic receptors. For example, even a short-term exposure (eg. 4 days) to continued adrenergic stimulation can cause down-regulation of β-receptors [47] and similar may occur during feed restriction. NEFA mobilisation and response is an exchange between the amount of body fat stores, energy intake and the differences in catecholamine sensitivity of fat stores [11]. The mean subcutaneous fat depths in the two experiments were very similar and indicate similar body fat reserves. Therefore, the mechanism behind the lower NEFA response in Experiment One may be simply be less time off feed, that all readily mobilised lipids were mobilised prior to sampling or an attenuation of β-adrenergic receptors and changes in catecholamine sensitivity in these cattle [11, 48].

Plasma CK and AST activates were the markers with the highest negative correlations with muscle glycogen, confirming our understanding around physical exertion and muscle glycogenolysis [1, 18]. Overall, the mean plasma CK activities were very high and the large range from 113.30 – 9384.90 IU/L suggests that muscle trauma was greater in some cattle than in others [33]. On principal component analyses, plasma CK and AST activities were co-directional with the plasma Na and Cl concentrations, both of which are indicative of hydration, plasma implying dehydrated cattle had greater muscle damage. While dehydration to some degree is an unavoidable consequence of transporting cattle long distances due to time off water and feed, this can be further exacerbated by exercise. Poor hydration prior to exercise in humans has been associated with poor skeletal muscle function and exacerbating muscle damage, with increased circulating CK and AST activities [49]. Thus, this finding highlights the importance of good hydration on muscle glycogen and possibly muscle or other tissue damage.

Plasma Cp concentrations were positively correlated with plasma glucose concentrations and on principal component analysis both the positive acute phase proteins HP-T and Cp were on the same plane as L-lactate and glucose. Acute stress has been associated with an acute phase reaction in cattle [50]. However, as there was no correlations between circulating Cp concentrations with muscle glycogen content or between plasma HP-T with any other biomarker the usefulness of these as indicators of stress at slaughter is questionable. Plasma Cp concentration is also a marker of body

copper status and increased concentrations are seen with injectable copper supplementations, a common management practices in copper deficient regions of southern Australian [51].

There were no significant correlations (r2 >0.1) between plasma magnesium concentration and any circulating biomarker, muscle glycogen or pHu. This was unexpected as the range in plasma magnesium concentrations suggested that at least some of the cattle were hypomagnesaemic [33]. Magnesium is reported to reduce the catecholamine effect during acute stress [52, 53] as well as reduce neuromuscular stimulation via its antagonism of calcium [54, 55]. Catecholamine release from activation of the sympathetic nervous system decreases plasma magnesium concentrations [56-58]. While the magnitude of β-adrenergic effect on magnesium depletion is unknown, it is suggested to be a bidirectional mechanism with hypomagnesaemia itself thought to activate catecholamine release. These data suggest the limited usefulness of plasma magnesium concentration as a marker of stress at slaughter. While plasma magnesium concentration provides an approximation of magnesium status where hypomagnesaemia is a reliable indicator of deficiency, hypomagnesaemia is not necessarily present in a magnesium-deficient state [54, 59]. Urinary magnesium excretion has a linear relationship with magnesium uptake in ruminants [60] and further research is required to see if this may be a more useful biomarker than peripheral magnesium concentrations.

Sample anticoagulant type, stability during storage and assay used for analysis is important to consider when assessing biochemical measurements. The greatest effect on stability is temperature, where non-refrigerated (>4oC) samples may artificially affect blood biomarkers from continued metabolism or hemolysis [61-63]. Studies evaluating freeze-thaw or long term frozen storage have shown that the biochemical markers used in this study, glucose, L-lactate, NEFA, BHB, CK, AST, sodium, chloride, magnesium, HP-T and Cp are stable when stored frozen [63-68]. NEFA is the biomarker which has previously been considered to be unstable during storage, particularly when heparin is used as an anticoagulant [61, 69]. Heparin is known to stimulate lipoprotein lipase, the major enzyme responsible for hydrolysing triglycerides to release free fatty acids [70, 71]. However as lipoprotein lipase is primarily bound to capillary endothelial cells, a heparin induced lipolytic effect is only seen under intravenous administration of heparin [72]. Baseline NEFA levels have been demonstrated to vary depending on anticoagulant and analysis type [61, 67-69, 73, 74], however the variation is small [73, 74] and in analysis of bovine NEFA, Stokol et al [67] commented that at 5% is was not clinically relevant. Using the Wako method of analysis, as used in our experiments, Stokol et al [67] observed that NEFA samples were stable up to 72 hours when stored at 4oC regardless of anticoagulant type. Stewart [68] also demonstrated stability of ovine NEFA in when chilled and using the Wako method of analysis. Stokol et al [67] observed no change in bovine NEFA if separation from whole blood was delayed for 24 hours but kept at 4oC, and frozen serum had only minor fluctuations (5%) over 28 days. The results of these studies suggest that if sampling methods, temperature and analysis technique are controlled, as per our experimental methodology, then the stability of NEFA and the other biomarkers assessed should remain stable between sampling and analysis.

4.2. The effect of Mixing and Transport Treatment on blood biomarkers

Contrary to our hypothesis, the activity of circulating enzymes that are indicative of muscle damage, CK and AST did not differ between transport methods. However, there was a small

significant between the mixing groups such that the plasma CK and AST activities of mixed heifers were higher than all other groups. While, AST is released from tissue following muscle damage, the enzyme is not tissue specific and may also indicate liver damage and thus must be interpreted along with plasma CK to be a reliable indicator of primary muscle degeneration [33]. Since CK is only released from myocardial and skeletal muscle, it is regarded as the most specific indicator of muscle degeneration [33]. While mixing had no significant effect on plasma CK activity in the present study, the physiological interpretation is linked with plasma AST activity and can extrapolated from previous CK research. Previous transport and mixing studies have measured CK alone, and there are no previous experiments including AST [75-77]. There was no differentiation between transport and mixing for CK, but the large increases of this enzyme indicate that that the experimental simulated commercial transport and behavioural interactions were resulting in marked physical activity and associated muscle damage. The elevated plasma level of CK rise in Experiment One is consistent with those reported by Buckham Sporer et al. [78], where truck transportation of young beef bulls resulted in a 221% increase of CK at 24 hours (1,229.72 ± 208.15 IU/L) post transit. Circulating CK activity is known to rise with transport and increasing stocking density [37, 79]. When unfamiliar bulls are mixed those displaying the greatest dominant agonistic behaviour pre-slaughter had higher peak circulating CK activity and a greater propensity towards having dark cutting carcasses [75-77]. While there are no published studies looking specifically at mixing of heifers, social regrouping studies surmised that increased mounting activity was the principle behaviour behind the increase in circulating CK activity [76]. Therefore, oestrus cycling of heifers may have compounded the social regrouping response in Experiment One. The mean plasma CK activity in Experiment One was twice observed in Experiment Two which may indicate that the muscle exertion and damage of cattle travelling on truck and boat was greater than truck and saleyard or may simply reflect half-life of the enzyme. Plasma CK has a relatively short half-life of approximately 4 hours and activity should return to normal within 2-3days unless myonecrosis persists [80]. Circulating AST on the other hand has a much longer half-life and activity can remain elevated in blood for 10days post injury [80], which suggests that overall the cattle in Experiment One experienced greater muscle damage as the range was higher in this cohort.

Contrary to our hypothesis, there was no difference between the mixing or transport groups in plasma glucose and L-lactate concentrations. This result supports findings by Polkinghorne et al. [38] who demonstrated no difference in circulating glucose or L-lactate concentrations between groups of cattle during extended road transport and McVeigh et al. [81] who demonstrated no difference in plasma glucose concentrations between mixed and non-mixed bulls. However, Warner et al. [4] demonstrated cattle subjected to electric cattle prodding 15minutes prior to slaughter had higher plasma L-lactate concentrations than unstressed cattle, although glucose was not measured in that study. The usefulness of plasma glucose and L-lactate as precise indicators of stress intensity may be limited as concentrations can be affected by various factors and plasma half-lives are relatively short [82]. Blood glucose has a relatively short half-life, as indicated by plasma glucose concentrations returning to normal approximately 90-120 minutes after a stress induced hyperglycaemic event [83]. Warris [37] found that transporting cattle caused a transient hyperglycaemia detectable at three hours but plasma glucose concentrations had returned to basal levels by six hours. Mudron et al. [84] evaluating the effect of surgical stress on blood glucose in dairy cows demonstrated that cows which

were in negative energy balance with hypoglycaemia prior to surgery had a reduced increase in blood glucose concentrations compared to normoglycaemic cows. Plasma L-lactate is derived from intense exercise and contraction linked increase in the rate of muscle glycogenolysis or from acute adrenergic stress where a surge in glycolysis can occur at two to three times the rate of oxidative phosphorylation and thus excess pyruvate is diverted to lactate [40, 41]. While the rate of clearance of plasma L-lactate in healthy cattle after varying levels of glycolysis does not appear to have been reported, in humans the plasma L-lactate half-life can be as short as 60 minutes if there is nothing that further impedes conversion back to pyruvate and full oxidation [85]. Experimental cattle were sampled 24 to 72 hours after consignment which suggests that the lack of differentiation between groups may have been that sampling missed the initial adrenergic surge. Interestingly, slaughter interval impacted plasma L-lactate concentrations in Experiment One where concentrations were higher in the rested cattle at 16.18 ± 0.42 mmol/L vs 13.36 ± 0.42 mmol/L, which suggests that glucose pre-cursors were repleted in the rested stock. However, the lack of difference between the groups in plasma glucose concentrations runs counter to this. Whilst the ability to differentiate between groups was low, the overall plasma glucose and L-lactate mean concentrations in these experiments were high, suggesting that cattle were experiencing high intensity stress. For example, plasma glucose concentrations were higher than previous studies testing pre-slaughter mixing (5.8 ± 0.9 mmol/L) [75] and transport stress (6.04 ± 0.035 mmol/L) [86]. However, the glucose concentrations observed in the present study were similar to those observed in a recent long distance transport stress in northern Australian (6.9 ± 0.9 mmol/L) [38] as well as a study investigating high rigour temperature in beef cattle (6.8 ± 0.9 mmol/L) [87].

While there were small changes in plasma βHB and NEFA concentrations, the only significant effects were between transport method and then only for one Ship or Saleyard. These results are consistent with recent beef transport studies where Buckham Sporer et al. [78] observed no change in circulating βHB concentrations at 24 or 48 hours post transit and Polkinghorne et al. [38] observed no change in plasma βHB or NEFA concentrations at 12, 24 or 36 hours post trucking and no correlations with either marker and muscle glycogen or pHu. The mean plasma βHB concentrations were higher than 0.19 ± 0.07 mmol/L reported by Polkinghorne et al. [38] but similar to Buckham Sporer et al. [78] of approximately 0.30 mmol/L (exact mean not stated, bar graph only). The mean plasma NEFA concentrations were similar to that reported by Kenny and Tarrant [76] after mixing stress (0.56 ± 0.04 mmol/L) and Polkinghorne et al. [38] (~0.43 mmol/L). The small and inconsistent response of plasma NEFA and βHB concentrations is consistent with previous studies and the lack of correlation with muscle glycogen content suggests that these energy substrates are not useful biomarkers for distinguishing between different pre-slaughter pathways.

Contrary to the hypothesis, there was little difference in plasma magnesium concentrations between the various pre-slaughter treatments. Mixing had a significant effect on plasma magnesium concentrations in Experiment One where steers that had never been mixed had a higher mean plasma magnesium concentrations (0.85 ± 0.03 mmol/L) than mixed steers (0.78 ± 0.03 mmol/L). While the impact of mixing on plasma magnesium concentrations was small in this study and not significant for any heifer groups, it is interesting to note that the heifers had lower mean concentrations than steers. Pre-slaughter magnesium supplementation is effective at reducing stress and improving meat quality in pigs and sheep [53, 88]. Therefore, these findings suggest that further research into pre-

slaughter magnesium supplementation for pasture raised beef could target heifers or those undergoing extended transport.

Contrary to the hypothesis, there was no consistent effect of treatments on plasma sodium and chloride concentrations. Plasma chloride concentration was elevated in only one treatment group, Experiment One cattle travelling on Ship-B, whereas there were no effects on plasma sodium concentrations. Sodium is the primary determinate of extracellular fluid volume and its concentration is regulated by changes in water intake and excretion. Sodium is the major extracellular cation and chloride the major extracellular anion, they are found in a 1.3 to 1 milliosmolar/liter ratio in plasma [89]. Water deprivation can cause a net water body deficit, dehydration of body tissues and increase in plasma osmolality which can be detectable in plasma as hypernatremia and hyperchloraemia. Plasma sodium and chloride concentrations were mostly within normal physiological ranges in the cattle in the present study, although the ranges indicate certain cattle were hypertonically dehydrated. Furthermore, isotonic dehydration can occur where losses of electrolytes and water are proportional thus there will be no shift in plasma sodium. Small shifts in body water to a loss of approximately 1-4% are not always clinically detectable and while there are multiple markers available to more precisely quantify dehydration often these are not practical in cattle, especially at an abattoir [86, 90]. It was interesting to note on principal component analysis the directional link with circulating muscle damage enzymes. Further work is required to quantify this impact measuring dehydration via multiple markers such as haematocrit, total protein, urine osmolality and blood urea nitrogen.

Plasma HP-T and Cp were not consistently responsive to the mixing or transport stressors applied in this experiment. Plasma HP-T concentrations were elevated only with one transport pathway in Experiment One and there was no effect of mixing or transport effects on Cp concentrations. The half-life of both enzymes is short, approximately 5 days, and it was expected that rested stock would have lower concentrations. While there was a significant effect of slaughter interval on plasma HP-T concentrations, the response was equivocal. In rested stock plasma Cp concentrations were elevated in Experiment One whereas they were lowered in Experiment Two. The acute phase proteins HP-T and Cp are an early defence mechanism released by the liver in response to stressors, infection or disease [91]. The influence of stress on the plasma concentration of these proteins has been varied in the bovine [91-93]. In a replicated transport and mixing study of weaner calves, Arthington et al. [93] observed no effect of mixing on acute phase proteins Cp or HP-T. Interestingly with transport there was an opposite effect between the two proteins where HP-T concentrations were higher in non-transported calves whilst Cp concentrations were higher in transported calves [93]. Physical stress in calves induced by different flooring types produced no change in circulating HP-T concentrations but an elevation of a different acute phase protein, amyloid-A [92]. As cortisol was also unchanged it is surmised that physical stress had no impact on plasma HP-T concentrations [92]. It is unclear why there was a varied response for acute phase proteins in the present experiment. Generally, there are basal concentrations of circulating Cp in unstressed cattle, whereas HP-T is often undetectable. Perhaps the simulated stressors were not specific enough to elicit a repeatable response. Further research is required to determine usefulness of these acute phase proteins, the results of the present experiment suggest they are not likely to be a useful objective measurement of transport of mixing stress.

Overall, plasma biomarkers failed to consistently distinguish between the radical differences in pre-slaughter mixing and transport implemented in the present study. We suggest that there may be multiple possible mechanisms behind this result. Certain markers, such as plasma glucose concentrations can change relatively quickly to differing stimuli and thus is can often be difficult to isolate the effects of experimental treatments from other effects in commercial studies. Secondly, the never-mixed control groups may have also experienced social re-ranking when they were separated into smaller groups on the day of consignment. Keeping the never mixed controls in smaller groups representing their stock-trailer groups would have provided for a more robust control. However, it is interesting to note that while some cattle were unloaded and reloaded onto trucks and multiple ships, which in theory should have been more stressful, this effect was undetectable. Thirdly, the strongest correlation with muscle glycogen content and pHu were with plasma CK and AST activities which suggests that the probable cause of muscle glycogen breakdown was the increased intracellular energy demand during muscle contraction, combined with β-adrenergic activation of glycogenolysis. Activation of the adrenergic system during exercise is much stronger when accompanied by emotional stress than it is without the additional stress [94]. This affirms the results by McVeigh et al. [95] who demonstrated that β-blockers administered to block adrenaline binding to muscle cells caused a reduction in the rate of glycogen depletion during the first hour of mixing stress, however overall these were ineffective at preventing overall depletion.

4.3. The effect of slaughter interval on plasma and muscle biomarkers

The results from this experiment affirmed the hypothesis that a two week rest period prior to slaughter was sufficient enough to replete muscle glycogen levels, although the effect was only significant in Experiment One. The rest period for Experiment Two corresponded with severe weather events in northwestern Tasmania including extreme cold temperatures, heavy rain and flooding. The plasma biomarkers showed little variation between the Experiment Two slaughter groups indicating the extreme weather events may have overwhelmed the variation imposed by the experimental stressors. There are a spectrum of responses to cold weather ranging from immediate psychological stress to neurological, hormonal and metabolic changes that act to reduce the rate of heat loss and restore lost heat [96]. Cold stress with shivering is known to increase resting metabolic rates and increase digesta passage rates which along with increased catecholamine secretion could have impaired glycogen repletion [97, 98]. Feed on offer in the holding paddocks at the processing plant was visually assessed by researchers as having an average of 1480 kg DM/hectare for Experiment One versus 725 kg for the Experiment Two. Pethick et al. [99] demonstrated the importance of a high energy diet for driving glycogen repletion, where a pasture hay diet of metabolisable energy 8 MJ/kg and crude protein 8% was insufficient to replete glycogen stores following exercise.

Abrupt short-term feed restriction impacts rumen microbial health [100] and activates acute phase protein response [101, 102] which can impact fermentation, feed intake and body weight post deprivation. Of importance to slaughter cattle after feed and water deprivation is body weight losses which have been approximately 5.7% after 24 hours [101] or 6.8% over 48 hours [103]. The impact of short term feed restriction and re-acclimatisation on feed intake and digestion is dependent on the

length and level of deprivation [104]. Furthermore the time to re-alimentation is positively related to pre-fasting feeding levels where greater intakes result in greater energy reserves and shorter post-fasting recovery [100]. Return to normal feed intake and body weight has been shown to take three days after a 24hour feed and or water restriction in beef heifers [101] and four days after a 48 hour deprivation [103]. However, Zhang [105] concluded severe feed restriction, to 25% of ration over 5 days, took two weeks for full voluntary feed intake to return. Time off feed was longer for Experiment Two which may have resulted in a longer post fast recovery and compounded ability for glycogenesis in the two week rest period.

Plasma CK activity was the only circulating biomarker where there was a significant effect of slaughter interval across both Experiments. There was a marked response in Experiment One, when directly killed stock exhibited a four-fold increase in plasma CK activity whereas in Experiment Two the increase was more modest (1.3 times). Interestingly, the mean plasma CK activity in the rested groups were higher in Experiment Two than Experiment One. As the half-life of CK is short, the persistently increased activity in Tasmanian rested stock may be secondary to increased shivering for thermogenesis during the extreme cold weather [106]. The magnitude of physical activity is a critical factor in muscle glycogen depletion [2]. This result suggests that cattle put through extensive pre-slaughter mixing stress may benefit from a two week rest period prior to slaughter to improve meat quality provided that the conditions are comfortable and cattle are well-fed. Implementation of such a strategy would require a cost benefit analysis and careful consideration around biosecurity. Pasture paddocks would require strict quarantine management to reduce the risk of parasite and other infectious disease contamination, and market specifications and assurance programs, such as the 100% pasture raised, hormone and antibiotic free cattle used in this experiment must be upheld.

5. Conclusions

The ability of pre-slaughter stressors to induce changes in plasma biomarkers sufficient to provide differences that may be used for future diagnostic purposes was limited in this experiment. Muscle damage as indicated by plasma CK activity was the biomarker most closely correlated with muscle ultimate pH and glycogen concentrations. A two week rest period under ideal conditions was effective at lowering this enzyme and improving muscle glycogen concentration. This suggests that contractile driven glycogen depletion was more important than acute adrenergic stress in this study and that the magnitude of physical activity was higher in females regardless of mixing group. The lack of effect of different social regrouping and transport pathways suggests the simulated stressors may not have been specific enough to elicit a repeatable response in biomarkers studied.

A positive finding from this experiment is that feed deprivation had no correlation with muscle pHu or glycogen and therefore long transport times, which are sometimes an unavoidable part of animal movement in large countries, should not impact meat quality.

Further research is required prior to commercial implementation, although this data suggests that plasma CK activity may be a useful objective measurement to distinguish cattle that may benefit from a rest period prior to slaughter. Environmental parameters and metabolisable energy intake must be carefully monitored during any pre-slaughter rest period to ensure conditions are optimal for muscle glycogen repletion as longer periods may be required to improve muscle glycogen concentrations.

Author Contributions: conceptualization and methodology, R.P., I.J.L., D.W.P., G.E.G., F.R.D., G.E.G., P.M. K.M.W.L.; formal analysis, G.T., K.M.W.L.; validation, K.M.W.L., G.T., I.J.L., D.W.P., G.E.G., R.P., F.R.D., P.M.; investigation, K.M.W.L., R.P., P.M., M.M.; resources, R.P., K.M.W.L.,P.M.,G.T.; writing—original draft preparation, K.M.W.L.; writing—review and editing, K.M.W.L., D.W.P., I.J.L., G.E.G., F.R.D.; G.T.; project administration, R.P., K.M.W.L., P.M.; funding acquisition, R.P., P.M., D.W.P.,F.R.D., I.J.L., G.E.G.,

Funding: This work was supported by the Meat and Livestock Australia (MLA, Sydney, New South Wales; Project Number L.EQT.1601 and L.EQT.1618. K.M.W.L. is a postgraduate student funded by MLA and Murdoch University.

Acknowledgments: The authors would like to thank the King Island beef producers and community for their continued support and assistance in animal management and data collection for this experiment. Thanks to the laboratory assistance of Diana Pethick at Murdoch University, the staff from MLA for their assistance with sample collection and technical support from Jota Grace Allison.

Conflicts of Interest: The authors declare no conflict of interest. The founding sponsors had no role in the design of the study; in the collection, analyses, or interpretation of data; in the writing of the manuscript, and in the decision to publish the results.

References

1. Tarrant, P., *Animal behaviour and environment in the dark-cutting condition in beef-a review.* Irish Journal of Food Science and Technology, 1989: p. 1-21.
2. Ferguson, D., H. Bruce, J. Thompson, A. Egan, D. Perry, and W. Shorthose, *Factors affecting beef palatability—farmgate to chilled carcass.* Australian Journal of Experimental Agriculture, 2001. **41**(7): p. 879-891.
3. Ferguson, D. and R. Warner, *Have we underestimated the impact of pre-slaughter stress on meat quality in ruminants?* Meat Science, 2008. **80**(1): p. 12-19.
4. Warner, R., D. Ferguson, J. Cottrell, and B. Knee, *Acute stress induced by the preslaughter use of electric prodders causes tougher beef meat.* Australian Journal of Experimental Agriculture, 2007. **47**(7): p. 782-788.
5. Chrousos, G.P., *Stressors, stress, and neuroendocrine integration of the adaptive response: The 1997 Hans Selye Memorial Lecture.* Annals of the New York Academy of Sciences, 1998. **851**(1): p. 311-335.
6. Selye, H., *The stress of life*. Vol. 5. 1956, New York: McGraw-Hill.
7. Johnson, E.O., T.C. Kamilaris, G.P. Chrousos, and P.W. Gold, *Mechanisms of stress: a dynamic overview of hormonal and behavioral homeostasis.* Neuroscience & Biobehavioral Reviews, 1992. **16**(2): p. 115-130.
8. Chrousos, G.P., D.L. Loriaux, and P.W. Gold, *Introduction: The concept of stress and its historical development*, in *Mechanisms of physical and emotional stress*. 1988, Springer. p. 3-7.
9. Moberg, G.P., *Biological response to stress: implications for animal welfare.* The biology of animal stress: basic principles and implications for animal welfare, 2000: p. 1-21.
10. Selye, H., *Forty years of stress research: principal remaining problems and misconceptions.* Canadian Medical Association Journal, 1976. **115**(1): p. 53.
11. Pethick, D., G. Harper, and F. Dunshea, *Fat metabolism and turnover*, in *Quantitative aspects of ruminant digestion and metabolism*, D. J., F. J.M., and F. J., Editors. 2005, CAB International: Wallingford UK. p. 345-372.
12. Seelig, M.S., *Consequences of magnesium deficiency on the enhancement of stress reactions; preventive and therapeutic implications (a review).* Journal of the American College of Nutrition, 1994. **13**(5): p. 429-446.
13. Murck, H., *Magnesium and affective disorders.* Nutritional neuroscience, 2002. **5**(6): p. 375-389.

14. Cannon, W.B., *Bodily Changes in Pain, Hunger, Fear and Rage: An Account of Recent Research Into the Function of Emotional Excitement, New York, Appleton-Century-Crofts. Professional Curriculum: A Call to Action.* Psychological Trauma: Theory, Research, Practice, and Policy, 1929. **1**(1): p. 3-23.
15. Kuchel, O., *Stress and catecholamines.* Methods and achievements in experimental pathology, 1991. **14**: p. 80.
16. Rooyackers, O.E. and K.S. Nair, *Hormonal regulation of human muscle protein metabolism.* Annual review of nutrition, 1997. **17**(1): p. 457-485.
17. Pethick, D. and J. Rowe, *The effect of nutrition and exercise in carcass parameters and the level of glycogen in skeletal muscle of Merino sheep.* Australian Journal of Agricultural Research, 1996. **47**(4): p. 525-537.
18. Gardner, G., B. McIntyre, G. Tudor, and D. Pethick, *The impact of nutrition on bovine muscle glycogen metabolism following exercise.* Australian Journal of Agricultural Research, 2001. **52**(4): p. 461-470.
19. McVeigh, J. and P. Tarrant, *Glycogen content and repletion rates in beef muscle, effect of feeding and fasting.* The Journal of nutrition, 1982. **112**(7): p. 1306-1314.
20. Knee, B., L. Cummins, P. Walker, G. Kearney, and R. Warner, *Reducing dark-cutting in pasture-fed beef steers by high-energy supplementation.* Australian Journal of Experimental Agriculture, 2007. **47**(11): p. 1277-1283.
21. Immonen, K., M. Ruusunen, K. Hissa, and E. Puolanne, *Bovine muscle glycogen concentration in relation to finishing diet, slaughter and ultimate pH.* Meat Science, 2000. **55**(1): p. 25-31.
22. Knee, B., L. Cummins, P. Walker, and R. Warner, *Seasonal variation in muscle glycogen in beef steers.* Australian Journal of Experimental Agriculture, 2004. **44**(8): p. 729-734.
23. MLA. *Meat Standards Australia beef information kit*. 2018; Available from: https://www.mla.com.au/globalassets/mla-corporate/marketing-beef-and-lamb/msa_tt_beefinfokit_jul13_lr.pdf (accessed on 24/06/2019).
24. Marti, S., R. Wilde, D. Moya, C. Heuston, F. Brown, and K. Schwartzkopf-Genswein, *Effect of rest stop duration during long-distance transport on welfare indicators in recently weaned beef calves.* Journal of animal science, 2017. **95**(2): p. 636-644.
25. Ferreira, G., C. Andrade, F. Costa, M. Freitas, T. Silva, and I. Santos, *Effects of transport time and rest period on the quality of electrically stimulated male cattle carcasses.* Meat science, 2006. **74**(3): p. 459-466.
26. Knowles, G., P. Warriss, S. Brown, and J. Edwards, *Effects on cattle of transportation by road for up to 31 hours.* The Veterinary Record, 1999. **145**(20): p. 575.
27. Loudon, K., I. Lean, D. Pethick, G. Gardner, L. Grubb, A. Evans, and P. McGilchrist, *On farm factors increasing dark cutting in pasture finished beef cattle.* Meat Science, 2018. **144**(Quality and Integrity for Global Consumers): p. 110-117.
28. Chan, T.M. and J.H. Exton, *A rapid method for the determination of glycogen content and radioactivity in small quantities of tissue or isolated hepatocytes.* Analytical biochemistry, 1976. **71**(1): p. 96-105.
29. Noll, F., *L+ lactate determination. In 'Methods of enzymatic analysis'.(Ed. HU Bergmeyer) pp. 583–588*. 1985, VCH Verlagsgesellschaft: Weinheim, Germany.
30. R Core Team, *A language and environment for statistical computing. R Foundation for Statistical Computing, Vienna, Austria.* URL:(https://www. R-project. org), 2018.
31. Wickham, H., *ggplot2: elegant graphics for data analysis*. 2016: Springer.
32. Bates, D., M. Mächler, B. Bolker, and S. Walker, *Fitting linear mixed-effects models using lme4.* Journal of Statistical Software, 2014. **67**(1): p. 1-48.

33. Radostits, O.M., C.C. Gay, K.W. Hinchcliff, and P.D. Constable, *Veterinary Medicine - a textbook of the diseases of cattle, horses, sheep, pigs and goats*. 9th ed. 2000, Sydney: WB Saunders Co.
34. Eckersall, P., F. Young, C. McComb, C. Hogarth, S. Safi, J. Fitzpatrick, A. Nolan, A. Weber, and T. McDonald, *Acute phase proteins in serum and milk from dairy cows with clinical mastitis.* Veterinary Record, 2001. **148**(2): p. 35-41.
35. Trevisi, E., M. Amadori, S. Cogrossi, E. Razzuoli, and G. Bertoni, *Metabolic stress and inflammatory response in high-yielding, periparturient dairy cows.* Research in veterinary science, 2012. **93**(2): p. 695-704.
36. Laven, R., K. Lawrence, and C. Livesey, *The assessment of blood copper status in cattle: a comparison of measurements of caeruloplasmin and elemental copper in serum and plasma.* New Zealand Veterinary Journal, 2007. **55**(4): p. 171-176.
37. Warriss, P., *The handling of cattle pre-slaughter and its effects on carcass and meat quality.* Applied animal behaviour science, 1990. **28**(1): p. 171-186.
38. Polkinghorne, R., J. Philpott, and J. Thompson, *Do extended transport times and rest periods impact on eating quality of beef carcasses?* Meat science, 2018. **140**: p. 101-111.
39. Gruber, S., J. Tatum, T. Engle, P. Chapman, K. Belk, and G. Smith, *Relationships of behavioral and physiological symptoms of preslaughter stress to beef longissimus muscle tenderness 1.* Journal of Animal Science, 2010. **88**(3): p. 1148-1159.
40. Pang, D.S. and S. Boysen, *Lactate in veterinary critical care: pathophysiology and management.* Journal of the American Animal Hospital Association, 2007. **43**(5): p. 270-279.
41. Bakker, J., M.W. Nijsten, and T.C. Jansen, *Clinical use of lactate monitoring in critically ill patients.* Annals of intensive care, 2013. **3**(1): p. 12.
42. Hermansen, L., E. Hultman, and B. Saltin, *Muscle glycogen during prolonged severe exercise.* Acta Physiologica Scandinavica, 1967. **71**(2-3): p. 129-139.
43. Harman, N. and D. Pethick, *The effects of sustained exercise on gluconeogenesis, glycogenolysis and glycogen synthesis in Merino sheep.* Australian Journal of Agricultural Research, 1994. **45**(6): p. 1189-1202.
44. Van Soest, P.J., *Nutritional ecology of the ruminant*. Second Edition ed. 1994, New York: Cornell university press.
45. Murray, R.K., Granner, D.K., Mayes, P.A., and Rodwell, V.W., *Harper's biochemistry*. 24th Edition, ed. 24th. 1996, Stamford: Appleton & Lange.
46. Nelson, D.L., A.L. Lehninger, and M.M. Cox, *Lehninger principles of biochemistry*. 2008: Macmillan.
47. Dunshea, F. and R. King, *Responses to homeostatic signals in ractopamine-treated pigs.* British Journal of Nutrition, 1995. **73**(6): p. 809-818.
48. Sechen, S.J., F.R. Dunshea, and D.E. Bauman, *Somatotropin in lactating cows: effect on response to epinephrine and insulin.* American Journal of Physiology-Endocrinology and Metabolism, 1990. **258**(4): p. E582-E588.
49. Ozkan, I. and C.H. Ibrahim, *Dehydration, skeletal muscle damage and inflammation before the competitions among the elite wrestlers.* Journal of physical therapy science, 2016. **28**(1): p. 162-168.
50. Cooke, R. and D. Bohnert, *Bovine acute-phase response after corticotrophin-release hormone challenge.* Journal of animal science, 2011. **89**(1): p. 252-257.
51. Blakley, B. and D. Hamilton, *Ceruloplasmin as an indicator of copper status in cattle and sheep.* Canadian Journal of Comparative Medicine, 1985. **49**(4): p. 405.

52. Kietzmann, M. and H. Jablonski, *On the blocking of stress by magnesium-aspartate hydrochloride in the pig.* Praktische Tierarzt (Germany, FR), 1985.
53. D'souza, D., R. Warner, B. Leury, and F. Dunshea, *The effect of dietary magnesium aspartate supplementation on pork quality.* Journal of Animal Science, 1998. **76**(1): p. 104-109.
54. Ryan, M.F., *The role of magnesium in clinical biochemistry: an overview.* Annals of clinical biochemistry, 1991. **28**(1): p. 19-26.
55. Schonewille, J.T., *Magnesium in dairy cow nutrition: an overview.* Plant and soil, 2013. **368**(1-2): p. 167-178.
56. Rayssiguier, Y., *Hypomagnesemia resulting from adrenaline infusion in ewes: its relation to lipolysis.* Hormone and Metabolic Research, 1977. **9**(04): p. 309-314.
57. Terashima, Y., Y. Muzushima, R. Yamaoka, and H. Itoh, *Effects of intravenous infusion of noradrenaline on plasma levels of Ca, inorganic P and Mg in sheep fed high or low calcium diet.* Japanese Journal of Zootechnical Science (Japan), 1983.
58. Yano, H., C. Nagaoka, and R. Kawashimai, *Effects of intravenous glucose or epinephrine injection on plasma concentrations of potassium, magnesium and insulin in sheep.* Journal of nutritional science and vitaminology, 1984. **30**(6): p. 535-540.
59. Whang, R., *Magnesium deficiency: pathogenesis, prevalence, and clinical implications.* The American journal of medicine, 1987. **82**(3): p. 24-29.
60. Martens, H. and M. Schweigel, *Pathophysiology of grass tetany and other hypomagnesemias: implications for clinical management.* Veterinary Clinics: Food Animal Practice, 2000. **16**(2): p. 339-368.
61. McGann, A.M. and A.W. Hodson, *Delay in cell separation, storage and anticoagulants induced inaccuracies in measuring plasma non-esterified fatty acids.* Clinica chimica acta, 1991. **197**(3): p. 265-270.
62. Morris, J., J. Fernandez, A. Chapa, L. Gentry, K. Thorn, and T. Weick, *Effects of sample handling, processing, storage, and hemolysis on measurements of key energy metabolites in ovine blood.* Small Ruminant Research, 2002. **43**(2): p. 157-166.
63. Rogiers, V., *Stability of the long chain non-esterified fatty acid pattern in plasma and blood during different storage conditions.* Clinica Chimica Acta, 1978. **84**(1-2): p. 49-54.
64. Jones, D., *Stability and storage characteristics of enzymes in cattle blood.* Research in veterinary science, 1985. **38**(3): p. 301-306.
65. Brinc, D., M.K. Chan, A.A. Venner, M.D. Pasic, D. Colantonio, L. Kyriakopolou, and K. Adeli, *Long-term stability of biochemical markers in pediatric serum specimens stored at− 80 C: a CALIPER Substudy.* Clinical biochemistry, 2012. **45**(10-11): p. 816-826.
66. Reynolds, B., B. Taillade, C. Médaille, F. Palenché, C. Trumel, and H.P. Lefebvre, *Effect of repeated freeze-thaw cycles on routine plasma biochemical constituents in canine plasma.* Veterinary Clinical Pathology, 2006. **35**(3): p. 339-340.
67. Stokol, T. and D. Nydam, *Effect of anticoagulant and storage conditions on bovine nonesterified fatty acid and β-hydroxybutyrate concentrations in blood.* Journal of dairy science, 2005. **88**(9): p. 3139-3144.
68. Stewart, S.M., *The influence of acute stress and feed deprivation on the meat quality and intermediary metabolism of Australian lamb*. 2017, Murdoch University.
69. Gleeson, M., *Effect of heparin and storage on human plasma free fatty acid concentration.* Clinica chimica acta, 1987. **169**(2-3): p. 315-318.
70. Eckel, R.H., *Lipoprotein lipase.* New England Journal of Medicine, 1989. **320**(16): p. 1060-1068.

71. Goldberg, I.J., *Lipoprotein lipase and lipolysis: central roles in lipoprotein metabolism and atherogenesis.* Journal of lipid research, 1996. **37**(4): p. 693-707.
72. Yang, J.-Y., T.-K. Kim, B.-S. Koo, B.-H. Park, and J.-W. Park, *Change of plasma lipoproteins by heparin-released lipoprotein lipase.* Experimental & molecular medicine, 1999. **31**(2): p. 60.
73. Menéndez, L.G., A.L. Fernández, A. Enguix, C. Ciriza, and J. Amador, *Effect of storage of plasma and serum on enzymatic determination of non-esterified fatty acids.* Annals of clinical biochemistry, 2001. **38**(3): p. 252-255.
74. Jeevanandam, M., Y.-C. Hsu, L. Ramias, and W. Schiller, *A rapid, automated micromethod for measuring free fatty acids in plasma/serum.* Clinical chemistry, 1989. **35**(11): p. 2228-2231.
75. Warriss, P., S. Kestin, S. Brown, and L. Wilkins, *The time required for recovery from mixing stress in young bulls and the prevention of dark cutting beef.* Meat Science, 1984. **10**(1): p. 53-68.
76. Kenny, F. and P. Tarrant, *The behaviour of young Friesian bulls during social re-grouping at an abattoir. Influence of an overhead electrified wire grid.* Applied Animal Behaviour Science, 1987. **18**(3-4): p. 233-246.
77. Kenny, F. and P. Tarrant, *The reaction of young bulls to short-haul road transport.* Applied Animal Behaviour Science, 1987. **17**(3): p. 209-227.
78. Buckham Sporer, K., P. Weber, J. Burton, B. Earley, and M. Crowe, *Transportation of young beef bulls alters circulating physiological parameters that may be effective biomarkers of stress.* Journal of Animal Science, 2008. **86**(6): p. 1325-1334.
79. Tarrant, P., F. Kenny, D. Harrington, and M. Murphy, *Long distance transportation of steers to slaughter: effect of stocking density on physiology, behaviour and carcass quality.* Livestock Production Science, 1992. **30**(3): p. 223-238.
80. Parkinson, T., J. Vermunt, and J. Malmo, *Diseases of cattle in Australasia: a comprehensive textbook*. 2010: New Zealand Veterinary Association Foundation for Continuing Education.
81. McVeigh, J., P. Tarrant, and M. Harrington, *Behavioral stress and skeletal muscle glycogen metabolism in young bulls.* Journal of Animal Science, 1982. **54**(4): p. 790-795.
82. Moberg, G.P., *Biological response to stress: key to assessment of animal well-being?*, in *Animal stress*. 1985, Springer. p. 27-49.
83. Reineke, E.L., *Overview of Diabetes Treatment in Animals*, in *Nutritional and Therapeutic Interventions for Diabetes and Metabolic Syndrome*. 2012, Elsevier. p. 499-511.
84. Mudroň, P., J. Rehage, H. Sallmann, M. Höltershinken, and H. Scholz, *Stress Response in Dairy Cows Related to Different Blood Glucose.* Acta Veterinaria Brno, 2005. **74**(1): p. 37-42.
85. Vincent, J.-l., P. Dufaye, J. Berré, M. Leeman, J.-P. Degaute, and R.J. Kahn, *Serial lactate determinations during circulatory shock.* Critical care medicine, 1983. **11**(6): p. 449-451.
86. Stockman, C., T. Collins, A. Barnes, D. Miller, S. Wickham, D. Beatty, D. Blache, F. Wemelsfelder, and P. Fleming, *Qualitative behavioural assessment and quantitative physiological measurement of cattle naïve and habituated to road transport.* Animal Production Science, 2011. **51**(3): p. 240-249.
87. Warner, R., F. Dunshea, D. Gutzke, J. Lau, and G. Kearney, *Factors influencing the incidence of high rigor temperature in beef carcasses in Australia.* Animal Production Science, 2014. **54**(4): p. 363-374.
88. Gardner, G., B. McIntyre, G. Tudor, and D. Pethick, *Nutritional influences on muscle glycogen recovery following exercise in sheep and cattle.* Recent advances in animal nutrition in Australia, 2001. **13**: p. 145-151.
89. Guyton, A.C., *Textbook of medical physiology*. Eighth ed. 1991, USA: W.B. Saunders Company.

90. Thrall, M.A., G. Weiser, R. Allison, and T. Campbell, *Veterinary hematology and clinical chemistry*. 2012: John Wiley & Sons.
91. Cray, C., J. Zaias, and N.H. Altman, *Acute phase response in animals: a review.* Comparative medicine, 2009. **59**(6): p. 517-526.
92. Alsemgeest, S., I. Lambooy, H. Wierenga, S. Dieleman, B. Meerkerk, A. Van Ederen, and T.A. Niewold, *Influence of physical stress on the plasma concentration of serum amyloid-a (SAA) and haptoglobin (HP) in calves.* Veterinary Quarterly, 1995. **17**(1): p. 9-12.
93. Arthington, J., S. Eicher, W. Kunkle, and F. Martin, *Effect of transportation and commingling on the acute-phase protein response, growth, and feed intake of newly weaned beef calves.* Journal of Animal Science, 2003. **81**(5): p. 1120-1125.
94. Karki, N., *The urinary excretion of noradrenaline and adrenaline in different age groups, its diurnal variation and the effect of muscular work on it.* Acta physiologica Scandinavica. Supplementum, 1956. **39**(132): p. 1-96.
95. McVeigh, J. and P. Tarrant, *Effect of propranolol on muscle glycogen metabolism during social regrouping of young bulls.* Journal of Animal Science, 1983. **56**(1): p. 71-80.
96. Young, B.A., *Some physiological costs of cold climates.* 1975.
97. Young, B., *Cold Stress as it Affects Animal Production 1.* Journal of Animal Science, 1981. **52**(1): p. 154-163.
98. Haman, F., F. Péronnet, G.P. Kenny, D. Massicotte, C. Lavoie, and J.M. Weber, *Partitioning oxidative fuels during cold exposure in humans: muscle glycogen becomes dominant as shivering intensifies.* The Journal of physiology, 2005. **566**(1): p. 247-256.
99. Pethick, D., L. Cummins, G. Gardner, B. Knee, M. McDowell, B. McIntyre, G. Tudor, P. Walker, and W. Warner, *The regulation by nutrition of glycogen level in the muscle of ruminants.* Recent advances in animal nutrition in Australia, 1999. **12**: p. 145-151.
100. Cole, N. and D. Hutcheson, *Influence of prefast feed intake on recovery from feed and water deprivation by beef steers.* Journal of Animal Science, 1985. **60**(3): p. 772-780.
101. Marques, R., D. Bohnert, O. de Sousa, A. Brandão, T. Schumaher, K. Schubach, M. Vilela, B. Rett, and R. Cooke, *Impact of 24-h feed or water, or both, deprivation on feed intake, metabolic, and inflammatory response in beef heifers.* Translational Animal Science, 2018. **2**(suppl_1): p. S95-S95.
102. Carroll, J.A., R.R. Reuter, C.C. Chase Jr, S.W. Coleman, D.G. Riley, D.E. Spiers, J.D. Arthington, and M.L. Galyean, *Profile of the bovine acute-phase response following an intravenous bolus-dose lipopolysaccharide challenge.* Innate Immunity, 2009. **15**(2): p. 81-89.
103. Phillips, W., P. Juniewicz, and D. VonTungeln, *The effect of fasting, transit plus fasting, and administration of adrenocorticotropic hormone on the source and amount of weight lost by feeder steers of different ages.* Journal of animal science, 1991. **69**(6): p. 2342-2348.
104. Zhang, S., R. Albornoz, J. Aschenbach, D. Barreda, and G. Penner, *Short-term feed restriction impairs the absorptive function of the reticulo-rumen and total tract barrier function in beef cattle.* Journal of animal science, 2013. **91**(4): p. 1685-1695.
105. Zhang, S., J. Aschenbach, D. Barreda, and G. Penner, *Recovery of absorptive function of the reticulo-rumen and total tract barrier function in beef cattle after short-term feed restriction.* Journal of animal science, 2013. **91**(4): p. 1696-1706.
106. Meltzer, H.Y., *Plasma creatine phosphokinase activity, hypothermia, and stress.* American Journal of Physiology-Legacy Content, 1971. **221**(3): p. 896-901.

© 2019 by the authors. Submitted for possible open access publication under the terms and conditions of the Creative Commons Attribution (CC BY) license (http://creativecommons.org/licenses/by/4.0/).
